# Pacific bluefin tuna, *Thunnus orientalis*, exhibits a flexible feeding ecology in the Southern California Bight

**DOI:** 10.1371/journal.pone.0272048

**Published:** 2022-08-25

**Authors:** Elan J. Portner, Owyn Snodgrass, Heidi Dewar

**Affiliations:** NOAA Southwest Fisheries Science Center, La Jolla, California, United States of America; Universidad de Cadiz Facultad de Ciencias del Mar y Ambientales, SPAIN

## Abstract

Pacific bluefin tuna, *Thunnus orientalis*, migrates from spawning grounds in the western Pacific Ocean to foraging grounds in the California Current System (CCS), where they are thought to specialize on high energy, surface schooling prey. However, there has been substantial variability in estimates of forage availability in the CCS over the past two decades. To examine the foraging ecology of juvenile *T*. *orientalis* in the face this variability, we quantified the diet and prey energetics of 963 individuals collected in the Southern California Bight (SCB) from 2008 to 2016. Using classification and regression tree analysis, we observed three sampling periods characterized by distinct prey. In 2008, *T*. *orientalis* diet was dominated by midwater lanternfishes and enoploteuthid squids. During 2009–2014, *T*. *orientalis* consumed diverse fishes, cephalopods, and crustaceans. Only in 2015–2016 did *T*. *orientalis* specialize on relatively high energy, surface schooling prey (e.g. anchovy, pelagic red crab). Despite containing the smallest prey, stomachs collected in 2009–2014 had the highest number of prey and similar total energetic contents to stomachs collected in 2015–2016. We demonstrate that *T*. *orientalis* is an opportunistic predator that can exhibit distinct foraging behaviors to exploit diverse forage. Expanding our understanding of *T*. *orientalis* foraging ecology will improve our ability to predict its responses to changes in resource availability as well as potential impacts on the fisheries it supports.

## Introduction

Pacific bluefin tuna, *Thunnus orientalis*, migrates from spawning grounds in the western Pacific at ages 0–3 years to forage in the California Current System (CCS) before returning west at ages 3–7+ years [1, 2]. These juveniles, typically ranging from ~ 40–175 cm fork length (FL), support commercial and recreational fisheries in both the U.S. and Mexican exclusive economic zones (EEZs), with peak U.S. landings from the Southern California Bight (SCB) [3]. Within the CCS, *T*. *orientalis* migrates seasonally from as far south as Baja California, Mexico, in winter to as far north as Washington state in fall [1, 4]. Seasonal migrations have been correlated with variability in upwelling, chlorophyll-*a* concentrations, and Pacific sardine (*Sardinops sagax*, hereafter “sardine”) landings in the CCS, though the latitudinal extents of migrations are variable and may be limited by size-specific thermal tolerance [1, 5]. Temperature can also constrain vertical movements, with dive duration and maximum depth generally increasing with ambient temperature [5, 6]. While physiology may limit maximum dive depth and duration, diving behaviors are likely influenced by the abundance and vertical distribution of forage [5, 7].

Although general movement patterns have been identified, there is a high degree of variability in the annual number of *T*. *orientalis* that migrate to the CCS as well as its seasonal distribution of within the CCS [1, 2, 4, 8]. Periods with low catch rates of *T*. *orientalis* in the eastern Pacific are linked to low numbers of migrants from the western Pacific [8, 9]. In some years, T. *orientalis* is more abundant in the Mexican EEZ, making them inaccessible to U.S. commercial vessels. In addition, fish foraging deeper in the water column are more difficult to capture for both commercial and recreational fishers. Linking variability in diet to forage availability will help to elucidate the drivers of horizontal and vertical movements of *T*. *orientalis*, and ultimately its availability to fishers.

Bluefin tunas (*T*. *orientalis*, *T*. *thynnus*, *and T*. *maccoyii*) in all ocean basins are considered to specialize on high energy, near-surface schooling prey (e.g., anchovies, herring) [10–12]. The availability of these prey can alter the timing and location of bluefin migrations. In the northwestern Pacific Ocean, migration of juvenile *T*. *orientalis* to feeding grounds in the CCS is delayed when landings of Japanese sardine (*Sardinops melanosticta*) are high [8]. Even when preferred prey are abundant, prey size structure can also impact predator movements. During the 1990’s and 2000’s the population of Atlantic herring, *Clupea harengus*, boomed in the Gulf of Maine, but landings of Atlantic bluefin tuna (*T*. *thynnus*) remained relatively low [13, 14]. Herring in the Gulf of Maine were anomalously small, requiring increased handling time and energetic expenditure per gram of prey, and *T*. *thynnus* moved north into Canadian waters where larger herring could be found [13]. Thus, prey abundance and size structure, as well as feeding energetics contribute to the distribution of bluefin tunas.

Over the past six decades (1960–2020) there has been substantial variability in forage availability in the CCS [15–17], but only two studies examined the diet of *T*. *orientalis* in the southern CCS during this period. Pinkas (1971) sampled in the 1960s, a period of extremely high northern anchovy abundance (*Engraulis mordax*, hereafter “anchovy”), and described diets dominated by anchovy. Conversely, Madigan et al. (2015) described moderate specialization on sardine during 2008–2010, a period with almost no anchovy and declining sardine abundance [17]. However, both studies were limited in sample size or duration and *T*. *orientalis* has remained in the SCB over the past two decades, including periods during which both anchovy and sardine abundances were low [16, 17]. Quantifying diets under variable conditions is crucial for understanding the effects of resource availability on predator movements between and within habitats.

To improve our understanding of the feeding ecology of *T*. *orientalis* in the CCS across diverse forage conditions, we examined the diets of individuals collected in the SCB from 2008 to 2016. The goals of this study were to: 1) describe the composition, size structure, and energetic value of prey; 2) quantify variability in foraging ecology over our study period; and 3) discuss how prey characteristics may impact foraging behaviors and ultimately, availability of *T*. *orientalis* to fishers. This work demonstrates the importance of longer-term diet monitoring by revealing an undescribed flexibility in *T*. *orientalis* foraging ecology not previously captured by shorter duration studies.

## Materials and methods

### Stomach collection, processing, and specimen morphometrics

The research and collection of *Thunnus orientalis* was conducted under the State of California Department of Fish and Wildlife permit No. SC-12372 issued to the National Oceanic and Atmospheric Administration’s Southwest Fisheries Science Center (NOAA SWFSC).

**Fig 1:**
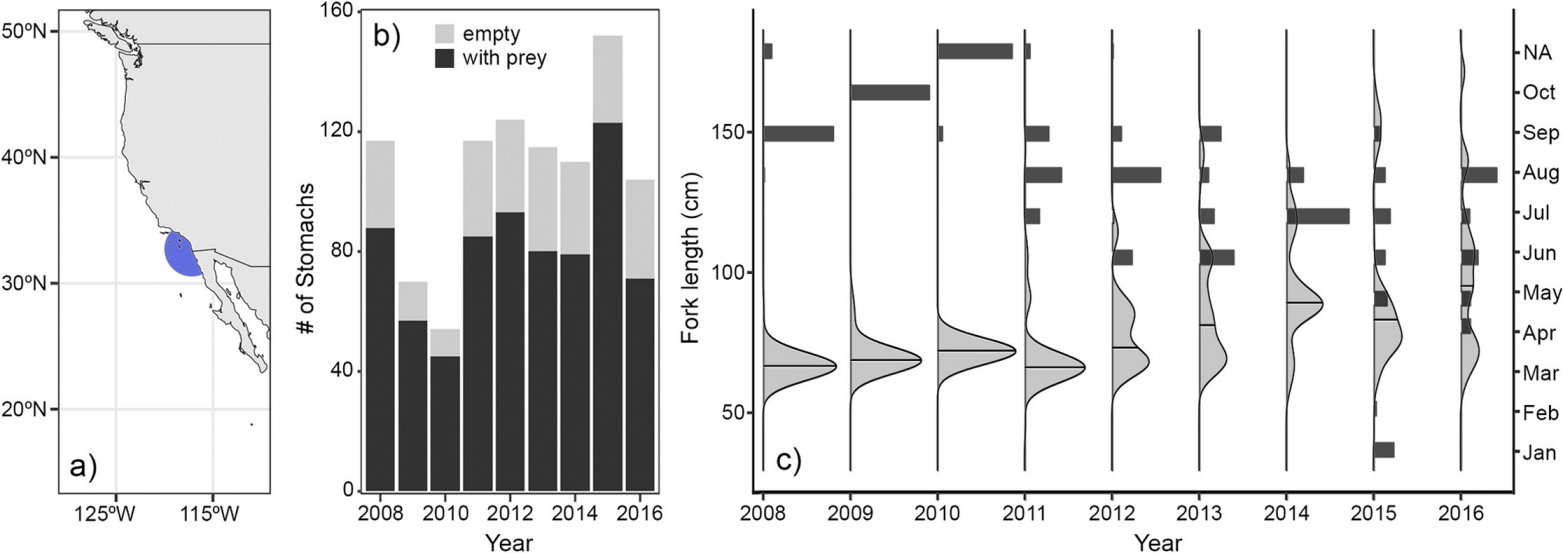
Summary of *Thunnus orientalis* sampling. (a) Map of study area showing sampling region in the Southern California Bight within ~150 nautical miles of San Diego, CA in blue. (b) Number of *T*. *orientalis* stomachs processed per year. (c) Month of collection (proportional bars, dark grey) and tuna fork length (density plots, light grey) for each year of sampling.

*T*. *orientalis* stomachs were collected from 2008 to 2016 between January and October (76% between June and September). Individuals were captured using rod and reel with hook and line by recreational anglers on commercial passenger fishing vessels and private boats. Specimens from at least 121 unique trips were donated by recreational fishers to the Southwest Fisheries Science Center and frozen at -20°C (S1 Table). Fishing trips operated in the SCB within 150 km of the port of San Diego (Fig 1A). Fork length (FL) and operculum length (OL) in centimeters, as well as capture date and location were recorded for each specimen when available (Fig 1). All fishes were captured during daylight hours, but time of capture was not reported. Capture locations were reported as coordinates (decimal degrees) or the colloquial names of common fishing grounds, which were then converted to approximate coordinates. For specimens where only the head and stomach were donated, FL was estimated from OL [18].

Carcasses were thawed overnight before we removed the stomachs and rinsed contents through a 0.5 mm brass mesh sieve. Stomach contents were stored in 70% ethanol until they could be identified to the lowest possible taxonomic level using published keys [19–22] and enumerated. No prey items were individually weighed, as most of the prey were at least partially digested and often represented only by hard parts (such as fish bones and cephalopod beaks). Only a single family of hyperiid amphipods was reliably identified (Phronimidae) and was increasingly identified as processor skill increased, so all hyperiid amphipods were grouped by suborder in our analyses (Hyperiidea). Other prey not identified at least to family were combined and considered per broad taxonomic grouping (fishes, cephalopods, and crustaceans). Bait used by fishers (fresh *S*. *sagax* and *E*. *mordax* >110 mm SL) and stomachs without prey were excluded from all analyses.

### Diet analysis

Prey counts were grouped by family and transformed into proportional abundances per stomach prior to diet similarity analyses. This approach reduces the influence of prey with high abundance but low frequency of occurrence and minimizes the effects of predator size on overall prey importance. Proportional prey abundance (*p*) is calculated by dividing the number of prey per group in an individual stomach by the total number of prey across all prey groups in that same stomach. Only prey families that contributed more than 1% mean proportional abundance ($\bar{p}$) across all stomachs were included in the similarity analyses. Families contributing less than 1% $\bar{p}$ were lumped together into an “Other” prey group per broad taxonomic grouping (e.g. “fishes” and “cephalopods”).

Tunas have been shown to exhibit diet variability with respect to size, season, capture location, and environmental conditions [12, 23, 24]. To examine the effects of collection year, month, location, and individual FL on *T*. *orientalis* diet, we performed classification and regression tree (CART) analysis in the *diet* package in R [25]. Collinearity among explanatory variables was examined using the *corrplot* package [26] (S1 Fig). CART analysis, described in Breiman et al. (1984), is a non-parametric modeling approach with applications to diet analyses extended by Kuhnert et al. (2012). By partitioning data through successive splitting that seeks to minimize an error criterion, the algorithm identifies optimal splitting values for each explanatory variable without requiring *a piori* knowledge. This allows for empirical partitioning of samples into groups with similar diets (e.g. tuna size classes) as well as non-linear relationships between explanatory variables and diet composition (e.g. variability in tuna size class definitions under different conditions). Additionally, CART analysis can accommodate missing covariate values by splitting on “surrogate” variables that have high correlation to the primary split [27]. These surrogates are also used to calculate the importance of each covariate relative to the selected variable at each split, which allows for recognition of covariates that are good predictors but masked from the final tree. Model performance was assessed using ten-fold cross validation and the final model was selected using the “1-SE rule” [28].

Terminal nodes of the CART analysis were used to inform further analyses on groups of *T*. *orientalis* with distinct diets. Diet composition was also summarized across terminal nodes of the CART analysis as the percent frequency of occurrence [*%FO = (number of stomachs containing prey*_*i*_ /total number of stomachs)*100] and the percent numerical abundance *[%N = (number of prey*_*i*_ /total number of prey) *100] of each prey type to facilitate comparison with previous studies.

Diet diversity and sample coverage were quantified with diversity accumulation curves using the *iNEXT* package in R [29]. Diet diversity is represented as the Shannon Index (*qD*, Hill number of order *q* = 1) [30]. Diet similarity within and among CART-defined groups was quantified using pairwise permutational multivariate analysis of variance (PERMANOVA) in Primer v7 [31]. Homogeneity of multivariate dispersion among groups was quantified using PERMDISP in Primer v7, a multivariate analog to Levene’s test for homogeneity of variance [31]. Principal coordinate analysis (PCoA) was performed in the *labdsv* package in R [32] to visualize the relative contributions of variable means and variances to diet differences among CART groups. Unless otherwise indicated, descriptive statistics are reported as means (± SD).

### Prey size and energetics

Lengths were measured for fresh and partially digested prey when possible and reported in mm as standard length (SL) for fishes, mantle length (ML) for cephalopods, and total length (TL) for crustaceans. Whole lengths from partial remains (teleost vertebrae, cephalopod beaks, and crustacean carapaces) were estimated following Glaser *et al*. [33] using published regressions (S2 Table). All cephalopod MLs were estimated from beaks.

Estimates of total stomach content mass and energetic value required length measurements from all prey. Mean lengths of measured prey were applied hierarchically to unmeasured prey of the same taxon from individuals in: 1) the same stomach; 2) the same sampling year; or 3) the same CART group. Family-level mean lengths were similarly applied to family-level prey IDs. Mantle lengths of unidentified squids for which rostral lengths were directly measured were estimated as the average ML from all beak to body length regressions used for identified cephalopods (S2 Table). For unmeasured, unidentified prey in each group, annual or CART group mean lengths were applied.

To estimate the energetic value of individual prey items, prey lengths were first converted to estimated body mass (g) using published regressions (S2 Table). For cephalopod prey, body mass was estimated directly from beak lengths. The mass of unidentified squids for which rostral lengths were directly measured was estimated as the average mass from all beak to body mass regressions used for identified cephalopods. Body mass was converted to energetic content using published energy density values for prey species following Glaser *et al*. [34] (S2 Table). Genus- or family- level estimates were used when prey taxonomic resolution was low or species-specific energy estimates were unavailable. For prey not identified at least to family (17% of all prey), energetic values were estimated as annual or CART group-level averages within the relevant taxonomic group and the energetic value of the total reconstituted mass within each stomach was quantified.

Comparisons of total content mass and energetic value among stomachs may be biased if contents represent different amounts of time since the last feeding event. Several studies have shown that bluefin tuna stomach fullness and prey digestion vary with time of day [12, 23, 35]. However, fullness is a metric based mostly on fresh prey and estimated peak feeding times are highly variable both within and across studies. Although we don’t know the exact capture times for any of our specimens, potential bias due to feeding time is reduced by considering the whole mass of both fresh and partially digested prey. Inclusion of reconstituted prey mass limits our temporal bias to the total gut clearance rate, which is also prey dependent and has been estimated at a minimum of 14–20 hours [36, 37]. However, the percent of empty stomachs was consistent across sampling years in this study (25 ± 5%, Fig 1B), suggesting a relatively consistent relationship between sampling times and gut clearance.

Our methods could also have overestimated total stomach content mass if mean prey lengths were not representative of unmeasured prey, or we included prey consumed on multiple days. The maximum reported daily rations for bluefin tunas in the size range sampled in this study are ~ 2–3% body mass (*BM*) [11, 12]. To minimize the chances that our methods for assigning masses to unmeasured prey overestimated prey mass or included prey from multiple days, we excluded stomachs with estimated contents > 3% *BM* from prey mass and energetic analyses.

### Comparing prey characteristics across bluefin sizes and CART groups

The size structure of *T*. *orientalis* in the SCB was variable across our study period and was reflected in the size structure of the tuna we sampled [3, 18]. To examine whether differences in stomach contents across years was impacted by predator size, we quantified the effects of *T*. *orientalis* mass on the total mass of prey per stomach as well as individual prey length using generalized additive models (GAMs) in the *mgcv* package [38]. GAMs were fit using restricted maximum likelihood parameter estimation and smoothing parameters for both models were estimated with Gamma distributions and “log” link functions.

Variability in *T*. *orientalis* foraging ecology across our time series was examined by comparing mean prey length, number, and energetic value per stomach among CART groups. None of these comparisons met both basic assumptions of normality and homoscedasticity for parametric mean comparisons, and non-parametric alternatives were used. When data variance was homoscedastic among groups but not normally distributed, a Kruskal-Wallis (KW) rank sum test was performed in R. If the global KW test statistic was significant, differences between group levels were quantified using pairwise Wilcoxon rank sum tests with a Bonferroni adjustment for multiple comparisons [39]. When data were both non-normal and heteroscedastic, a Welch’s ANOVA [40] was performed on ranks [41], and pairwise differences between ranks among groups were examined using Games-Howell *post hoc* tests in the *rstatix* package [42, 43].

The degree of specialization of *T*. *orientalis* diet was estimated by examining the prey-specific proportion (analogous to ‘prey-specific abundance’, [44]) of the most frequently consumed prey in each CART group. For each prey group found in at least 20% of stomachs (*prey*_*i*_), we quantified the mean proportion and mean number of *prey*_*i*_ in stomachs that contained *prey*_*i*_. A prey-specific proportion threshold of 0.5 was used to distinguish specialist (> 0.5) from generalist (< 0.5) foraging behaviors.

## Results

### Diet summary

Of the 963 *T*. *orientalis* stomachs sampled, 721 (75%) contained prey (Fig 1B). Specimens sampled in 2008–2010 were relatively small (61–93 cm FL, 5.43–10.14 kg), and larger individuals across a broader size range (42–174 cm FL, 1.83–102.54 kg) were sampled during 2011–16 (Fig 1C). A total of 21,189 prey items were identified across 48 groups (Table 1). Three crustacean groups, seven fish families, and seven cephalopod families contributed >1% $\bar{p}$ to *T*. *orientalis* diet across all years (Table 1). Of these 16 families, eight were represented by a single species, and four by a single genus. An additional 13 fish families and four cephalopod families contributed <1% $\bar{p}$ across all samples and were lumped into “Other fishes” and “Other cephalopods” groups, respectively.

**Table 1:** Diet composition of *Thunnus orientalis*. The percent number (%N), number, percent frequency of occurrence (%FO), and frequency are given for each prey item per CART-defined sampling period. Asterisks indicate prey families that were lumped into an “Other” prey group for diet similarity analyses.

		*%N (number)*			*%FO (frequency)*		
Family/Group	Prey ID	2008	2009–14	2015–16	2008	2009–14	2015–16
Cephalopods							
Argonautidae	*Argonauta* sp.	0.1 *(1)*	1.9 *(268)*	0.1 *(6)*	1.1 *(1)*	14.6 *(64)*	4.5 *(6)*
Enoplotethidae	*Abraliopsis* sp.	29.6 *(590)*	0.1 *(11)*	0.4 *(18)*	77.3 *(68)*	0.2 *(1)*	2.2 *(3)*
Gonatidae	*Gonatopsis* sp.	0.1 *(1)*	0.0 *(6)*	0.1 *(4)*	1.1 *(1)*	0.9 *(4)*	2.2 *(3)*
	*Gonatus* sp.	1.1 *(22)*	5.2 *(744)*	0.7 *(33)*	9.1 *(8)*	21.9 *(96)*	6.7 *(9)*
Loliginidae	*Doryteuthis opalescens*	0.8 *(15)*	3.2 *(466)*	0.4 *(20)*	5.7 *(5)*	14.6 *(64)*	9.0 *(12)*
Octopodidae	*Octopus bimaculatus*	-	0.0 *(3)*	0.0 *(1)*	-	0.2 *(1)*	0.7 *(1)*
	*Octopus rubscens*	-	3.8 *(544)*	0.2 *(9)*	-	15.9 *(70)*	2.2 *(3)*
Octopoteuthidae	*Octopoteuthis* sp.	0.1 *(2)*	4.3 *(625)*	-	2.3 *(2)*	21.6 *(95)*	-
Onychoteuthidae	*Onychoteuthis borealijaponica*	1.1 *(22)*	3.9 *(565)*	0.3 *(16)*	18.2 *(16)*	18.5 *(81)*	9.0 *(12)*
*Amphitretidae	*Japetella heathi*	0.1 *(2)*	0.1 *(21)*	-	2.3 *(2)*	2.1 *(9)*	-
*Cranchiidae	*Leachia* sp.	-	-	0.0 *(1)*	-	-	0.7 *(1)*
*Histioteuthidae	*Histioteuthis heteropsis*	0.1 *(2)*	0.0 *(5)*	-	1.1 *(1)*	0.7 *(3)*	-
*Ommastrephidae	*Dosidicus gigas*	0.8 *(16)*	0.3 *(39)*	-	10.2 *(9)*	2.7 *(12)*	-
Unidentified squids	Unidentified squids	1.9 *(37)*	16.6 *(2380)*	1.2 *(60)*	4.5 *(4)*	42.8 *(188)*	13.4 *(18)*
**Fishes**							
Carangidae	*Trachurus symmetricus*	0.1 *(1)*	10.5 *(1514)*	0.6 *(31)*	1.1 *(1)*	33.0 *(145)*	8.2 *(11)*
Clupeidae	*Sardinops sagax*	0.2 *(4)*	1.7 *(246)*	0.9 *(44)*	3.4 *(3)*	16.2 *(71)*	13.4 *(18)*
Engraulidae	*Engraulis mordax*	0.2 *(4)*	2.8 *(400)*	10.4 *(500)*	3.4 *(3)*	5.5 *(24)*	30.6 *(41)*
Myctophidae	*Ceratoscopelus townsendi*	5.1 *(101)*	0.0 *(6)*	0.6 *(27)*	43.2 *(38)*	0.2 *(1)*	1.5 *(2)*
	*Diaphus theta*	4.3 *(86)*	0.4 *(51)*	-	31.8 *(28)*	2.5 *(11)*	-
	*Nannobrachium ritteri*	0.2 *(3)*	0.2 *(31)*	0.0 *(1)*	3.4 *(3)*	1.4 *(6)*	0.7 *(1)*
	*Protomyctophum crockeri*	0.1 *(1)*	0.2 *(26)*	-	1.1 *(1)*	1.1 *(5)*	-
	*Stenobrachius leucopsaurus*	7.4 *(147)*	0.2 *(22)*	-	37.5 *(33)*	2.3 *(10)*	-
	*Symbolophorus californiensis*	-	0.3 *(36)*	0.0 *(1)*	-	3.2 *(14)*	0.7 *(1)*
	*Tarletonbeania crenularis*	-	0.0 *(7)*	-	-	0.2 *(1)*	-
	*Triphoturus mexicanus*	31.4 *(627)*	0.3 *(48)*	-	61.4 *(54)*	0.2 *(1)*	-
	Myctophidae	0.1 *(1)*	-	-	1.1 *(1)*	-	-
Scomberesocidae	*Cololabis saira*	0.2 *(4)*	2.0 *(294)*	0.2 *(11)*	3.4 *(3)*	13.0 *(57)*	3.7 *(5)*
Scombridae	*Scomber japonicus*	-	4.1 *(594)*	0.1 *(4)*	-	18.0 *(79)*	1.5 *(2)*
Sebastidae	*Sebastes* sp.	0.8 *(15)*	5.0 *(722)*	2.7 *(132)*	13.6 *(12)*	19.6 *(86)*	15.7 *(21)*
*Argentinidae	*Argentina sialis*	-	-	0.2 *(11)*	-	-	2.2 *(3)*
*Centrolophidae	*Icichthys lockingtoni*	-	0.0 *(1)*	-	-	0.2 *(1)*	-
*Exocoetidae	*Cheilopogon pinnatibarbatus*	-	-	0.0 *(2)*	-	-	1.5 *(2)*
*Labridae	*Oxyjulis californica*	-	-	0.1 *(3)*	-	-	1.5 *(2)*
*Merlucciidae	*Merluccius productus*	-	0.4 *(52)*	0.0 *(2)*	-	2.1 *(9)*	1.5 *(2)*
*Microstomatidae	*Nansenia* sp.	-	0.1 *(10)*	-	-	1.4 *(6)*	-
*Ophidiidae	*Chilara taylori*	-	0.1 *(20)*	0.3 *(16)*	-	2.1 *(9)*	7.5 *(10)*
*Paralepididae	*Lestidiops ringens*	-	0.1 *(21)*	-	-	1.8 *(8)*	-
	*Magnisudis atlantica*	-	0.0 *(1)*	-	-	0.2 *(1)*	-
*Pleuronectidae	*Pleuronichthys decurrens*	-	0.1 *(9)*	-	-	0.5 *(2)*	-
*Pleuronectiformes	Pleuronectiformes	-	0.0 *(4)*	-	-	0.5 *(2)*	-
*Sciaenidae	*Seriphus politus*	-	0.0 *(2)*	-	-	0.2 *(1)*	-
*Scopelarchidae	*Rosenblattichthys volucris*	0.1 *(2)*	-	-	1.1 *(1)*	-	-
*Syngnathidae	*Syngnathus californiensis*	0.2 *(4)*	0.2 *(25)*	0.0 *(1)*	2.3 *(2)*	2.5 *(11)*	0.7 *(1)*
Unidentified fishes	Unidentified fishes	1.9 *(37)*	1.7 *(243)*	1.4 *(67)*	26.1 *(23)*	17.5 *(77)*	17.9 *(24)*
**Crustaceans**							
Hyperiidea	*Phronima* sp.	-	1.9 *(280)*	1.0 *(50)*	-	11.6 *(51)*	3.0 *(4)*
	Hyperiidea	-	23.3 *(3354)*	1.5 *(74)*	-	47.8 *(210)*	4.5 *(6)*
Munididae	*Pleuroncodes planipes*	-	-	74.5 *(3591)*	-	-	84.3 *(113)*
Unidentified Malacostraca	Unidentified Malacostraca	12.4 *(247)*	4.7 *(678)*	1.8 *(85)*	59.1 *(52)*	20.3 *(89)*	12.7 *(17)*
	**Total**	**1994**	**14374**	**4821**	**88**	**439**	**194**

### Classification and regression tree analysis

**Fig 2:**
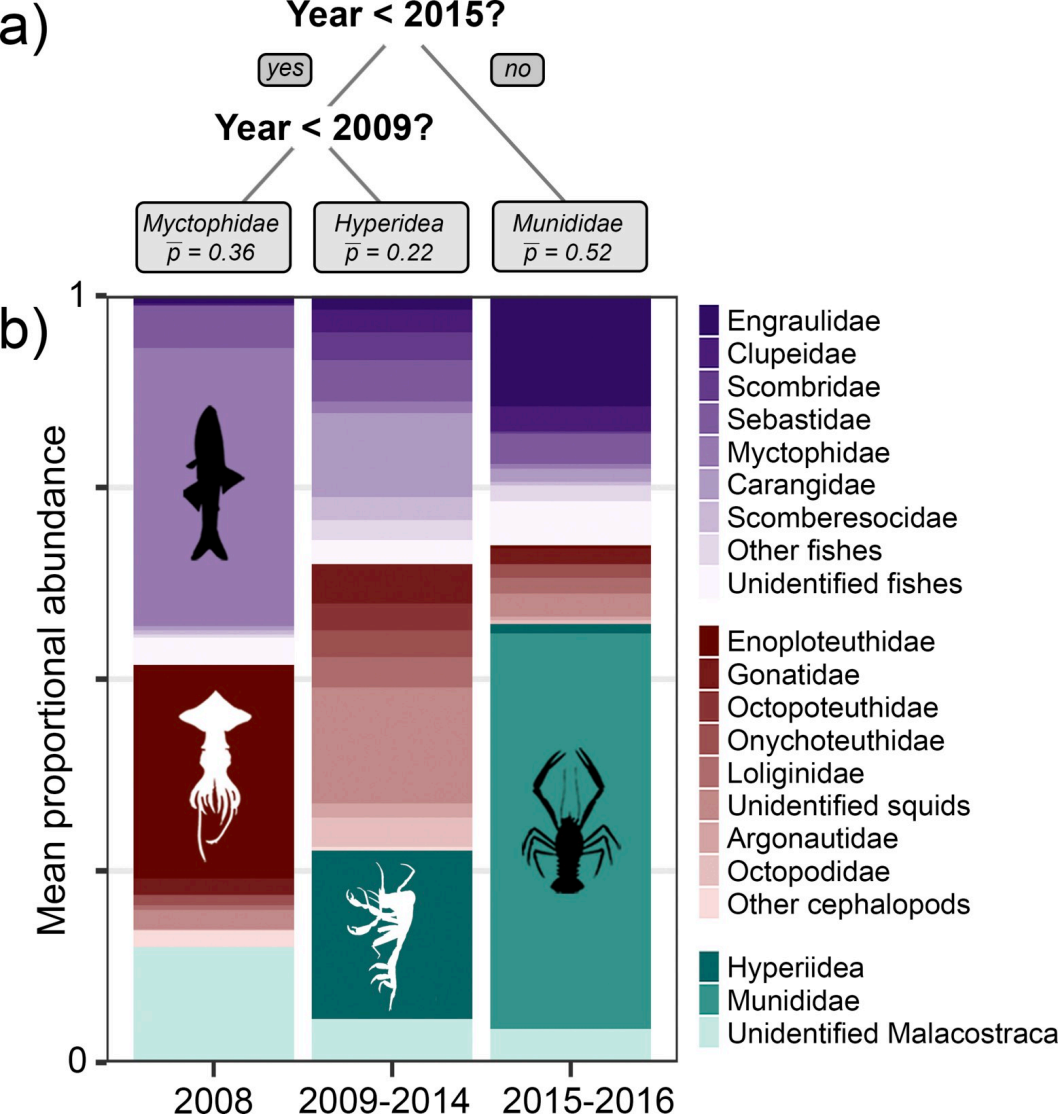
Classification and regression tree (CART) analysis describing groups of *Thunnus orientalis* with similar diets. (a) The selected tree, with splits based on responses to the condition listed at each branching point; left for affirmative, and right for negative. The mean cross validated proportion of the most abundant prey group is given below each terminal node. (b) Diet composition for prey contributing >1% mean proportional abundance in each CART group (purples = fishes, browns = cephalopods, and greens = crustaceans).

Stomach contents of *T*. *orientalis* were partitioned into three groups based on collection year: 2008 (*n* = 88), 2009–2014 (*n* = 439), and 2015–2016 (*n* = 194) (Fig 2A). The pruned classification tree had a cross-validated error rate of 0.225 (*SE* = 0.02, *R*^*2*^ = 0.78), and collection year was the most important predictor of diet (relative importance = 1). Although collection month was significantly correlated with year (Pearson’s *R* = -0.61, Figs 1C and S1), it was a poor surrogate for collection year in the CART analysis. Month of collection was largely uninformative (relative importance = 0.01), and neither latitude, longitude, nor specimen fork length were reliable predictors of diet in our dataset (relative importance = 0).

Fishes in the family Myctophidae and squids in the family Enoploteuthidae dominated the diet in 2008 ($\bar{p}$ = 0.36 and 0.29, respectively). During 2009–2014, *T*. *orientalis* consumed hyperiid amphipods ($\bar{p}$ = 0.22) and a relatively high diversity of fishes and cephalopods. The pelagic red crab, *Pleuroncodes planipes* ($\bar{p}$ = 0.52), and northern anchovy, *E*. *mordax* ($\bar{p}$ = 0.14), were dominant in 2015–2016 (Fig 2B and S3 Table).

### Diet variability among sampling periods

**Fig 3:**
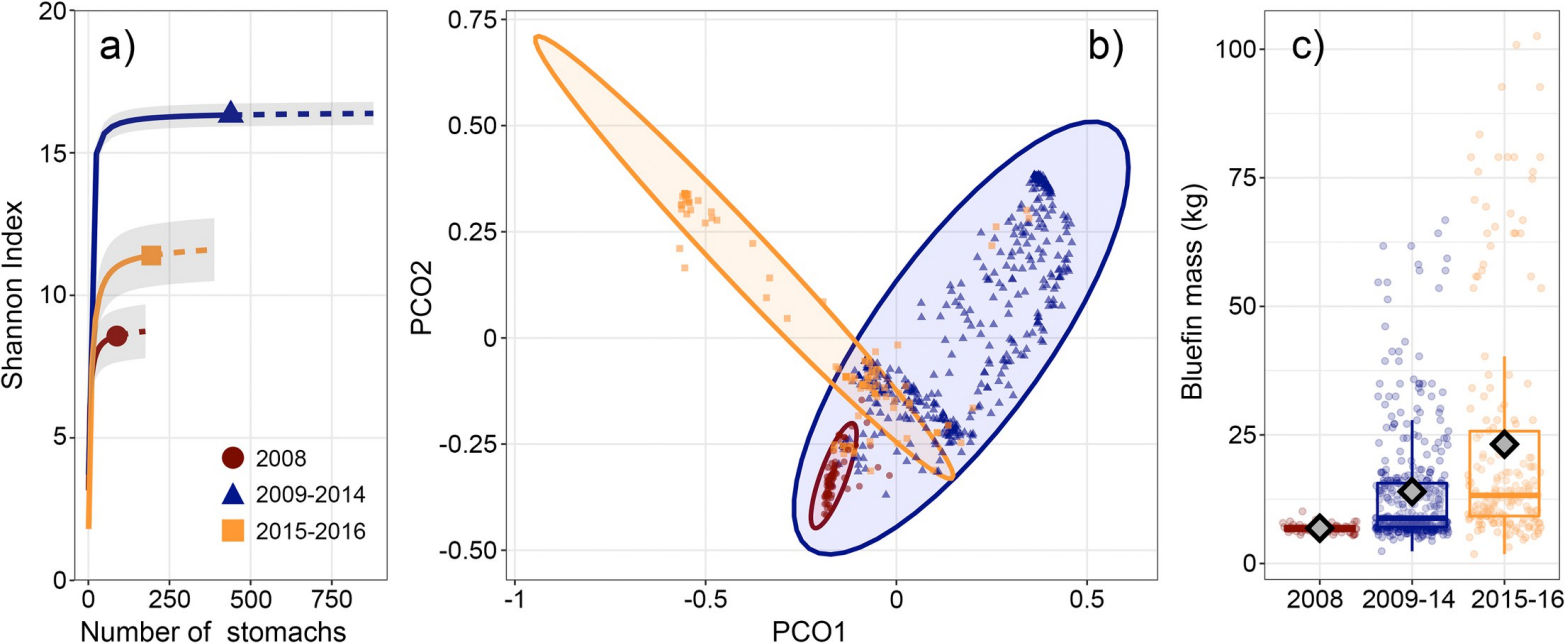
Differences in diet and *Thunnus orientalis* size structure among sampling periods. (a) Diversity accumulation curves for *T*. *orientalis* diets by CART group. Solid lines represent interpolated diversity and dashed lines represent extrapolated diversity to double the sample size of each group. The legend in panel (a) also applies to panel (b). (b) Principal coordinate analysis of *T*. *orientalis* diet by CART group. Ellipses represent 95% confidence intervals for each group. (c) Box plots of *T*. *orientalis* mass across sampling periods with means represented by grey diamonds on each boxplot (*n* = 644).

All CART groups reached 99% sample coverage; only 1% of the total expected family-level prey diversity remained unsampled in our dataset. Although diet diversity was highest in the CART group with the most samples and sampling years (2009–2014, *qD* = 16.33), samples collected during this period had relatively high prey diversity within individual stomachs and accumulated diversity at the highest rate (Fig 3A). Samples in the 2009–2014 group reached 99% sample coverage with only 35 stomachs compared to 88 and 135 stomachs needed to reach the same level of sample coverage in 2008 and 2015–2016, respectively (S4 Table).

Variability in diet composition within CART groups was significantly smaller than variability among groups (PERMANOVA, *t* = 8.87–10.27, *p* = 0.001). Diet similarity was highest within the 2008 group and lowest within the 2009–2014 group (mean pairwise similarity = 43.06% and 20.60%, respectively). Among group similarity was low for all groups (mean pairwise similarity = 3.13–4.70%). There was also significant variability in dispersion among groups (PERMDISP, Global *F* = 35.92, *p* = 0.001, pairwise *t* = 3.99–9.44, *p* < 0.005), and PCoA demonstrates that both the mean and variance of diet composition contributed to the observed differences among CART groups (Fig 3B).

### Effects of tuna size on stomach contents

Of the 721 *T*. *orientalis* with non-empty stomachs, FL and prey % *BM* could be estimated for 644 specimens. The estimated prey mass to predator mass ratio of 52 stomachs exceeded 3% *BM* and were excluded from prey mass and energetics analyses (7 stomachs from 2008, 30 from 2009–14, and 15 from 2015–16). The mean mass of prey in the remaining stomachs (*n* = 592) was 58.3 ± 68.7 g, representing 0.5 ± 0.6% *BM*, both within reported ranges of bluefin stomach content mass and daily ration for the sizes of tuna sampled [11, 12, 36]. *T*. *orientalis* mass varied among CART groups (Welch’s ANOVA on ranks, *F* = 183.04, *p* < 0.001, Fig 3C), but was not a useful predictor of estimated stomach content mass among sampling years (GAM, *Adj*. *R*^*2*^ = 0.04, Table 2 and S2 Fig).

**Table 2:** Summary of covariate contributions to generalized additive models describing the effects of tuna mass and sampling year on prey mass per stomach and prey length. Coefficient estimates (“estimate”), *t*-values, and *p*-values are given for each parametric coefficient. Estimated degrees of freedom (edf), *F*-values, and *p*-values are given for each smooth term.

*Total prey mass per stomach ~ s(tuna mass*, *k = 5) + s(year*, *k = 5)*, *n* = 592, *Adj*. *R*^*2*^ = 0.04			
**Parametric coefficients**	**estimate**	***t*-value**	***p***
*Intercept*	4.02	85.13	< 0.001
**Smooth terms**	**edf**	***F***	***p***
*s(tuna mass)*	1.37	0.66	0.14
*s(year)*	2.54	7.50	< 0.001
*Prey length ~ prey group + s(tuna mass*, *k = 5) + s(year*, *k = 5)*, *n* = 644, *Adj*. *R*^*2*^ = 0.22			
**Parametric coefficients**	**estimate**	***t*-value**	***p***
*Intercept*	1.15	64.02	< 0.001
*prey group*	-0.30–0.74	-6.11–20.21	< 0.001
**Smooth terms**	**edf**	***F***	***p***
*s(tuna mass)*	0.70	0.29	0.19
*s(year)*	3.95	60.45	< 0.001

After accounting for prey group and sampling year, *T*. *orientalis* mass was also not a useful predictor of prey length (GAM, *Adj*. *R*^*2*^ = 0.22, Table 2 and S3 Fig). Thus, additional analyses were performed under the assumption that variability in the mass of *T*. *orientalis* sampled during our study period was not a significant driver of variability in the quantity, size-structure, or composition of prey in a single stomach. We focused on examining differences in prey characteristics among CART groups, which were represented by distinct taxa.

### Prey size

**Fig 4:**
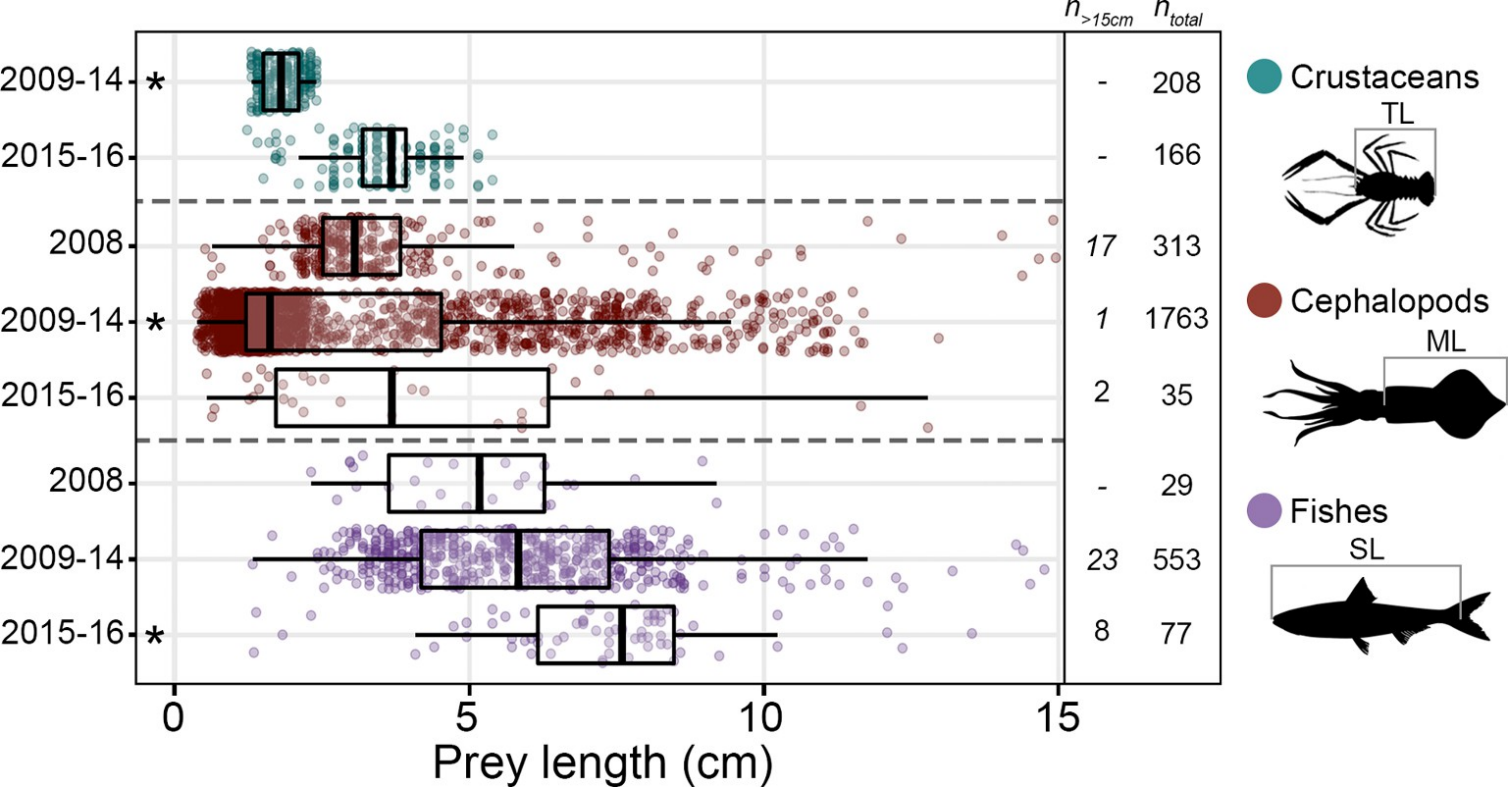
Lengths of crustacean, cephalopod, and fish prey consumed by *Thunnus orientalis* in the Southern California Bight, 2008–2016. Boxplots of prey length are given for each prey type measured per CART group. The x-axis was cropped at 15 cm to improve visualization, but the boxplots dimensions include all measured individuals. The number of individuals greater than 15 cm body length (*n*_*>15cm*_) as well as the total number of measured prey (*n*_*total*_) is provided. Within each prey type, asterisks indicate groups that are significantly different from all other groups. The color key and body measurements for each prey type are given at right.

A total of 3,144 prey were individually measured, with means of 6.86 (± 4.82) cm SL for fish, 3.29 (± 3.31) cm ML for cephalopod, and 2.58 (± 1.03) cm TL for crustacean prey. Cephalopod prey length ranks were significantly different among CART groups (Welch’s ANOVA on ranks, *F* = 180.88, *p* <0.001, Fig 4). The smallest individuals were consumed in 2009–14 (Games-Howell, *t* = 3.06–19.04, *p* < 0.01), but there was no difference in the length ranks of cephalopods consumed in 2008 and 2015–16 (Games-Howell, *t* = 1.14, *p* = 0.49). The lengths of fish and crustacean prey were also different among CART groups (Welch’s ANOVAs on ranks; *F* = 26.30, 555.76, respectively; *p* < 0.001). Both fish (Games-Howell, *t* = 5.71–6.67, *p* < 0.001), and crustacean (Games-Howell, *t* = 23.57, *p* < 0.001) length ranks were highest in 2015–16 when anchovies and pelagic red crabs were the dominant prey (Figs 4 and 2B).

### Prey number and energetics

**Fig 5:**
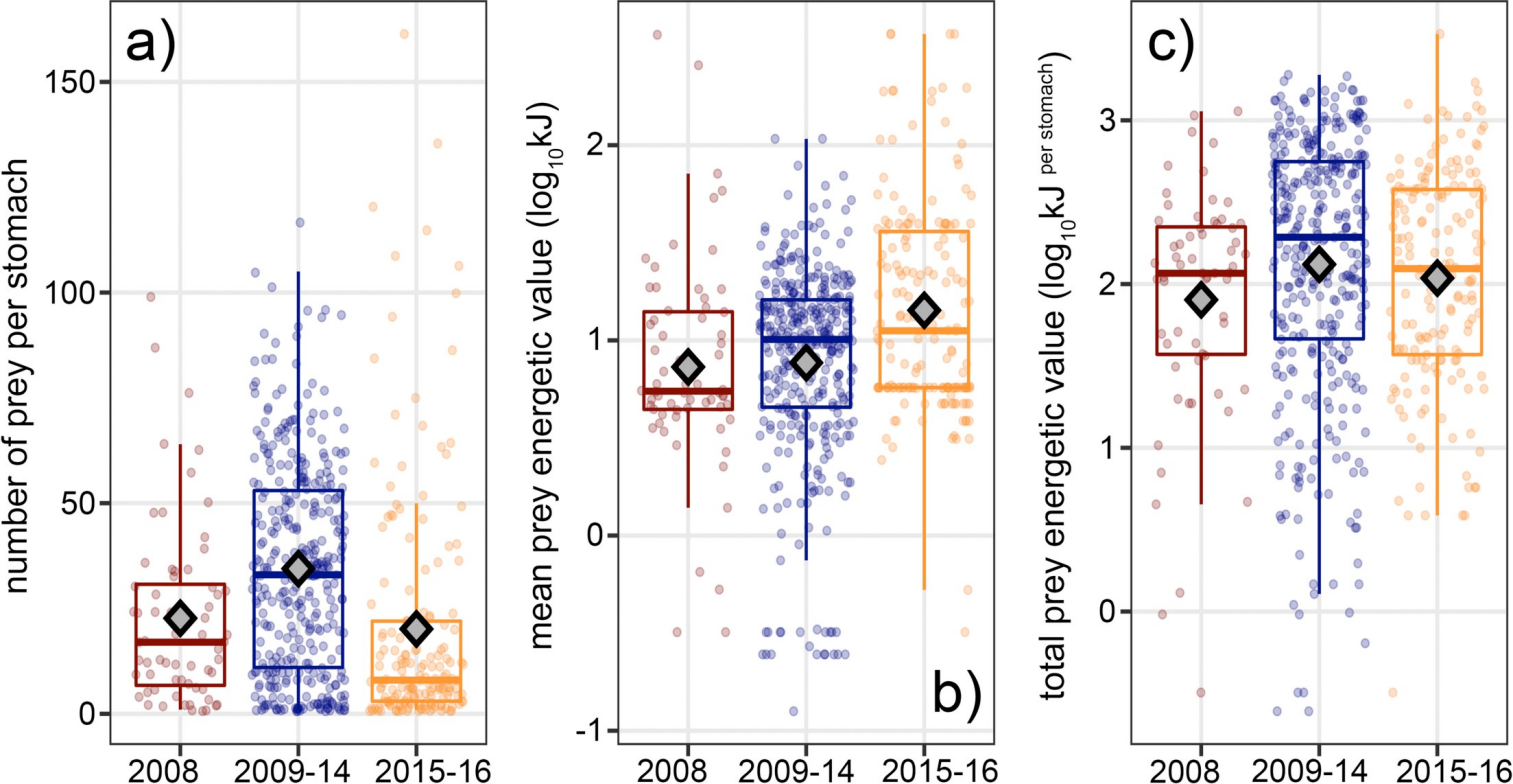
Characteristics of the stomach contents of *Thunnus orientalis*. In all panels, the mean values per CART group are given as grey diamonds. For each stomach with estimated prey mass less than 3% *BM* (*n* = 592, see Methods), the number of prey per stomach (a), mean energetic value of individual prey (b), and total energetic value of stomach contents (c) are given per CART group.

The total number of prey per stomach was different among CART groups (Welch’s ANOVA on ranks, *F* = 31.60, *n* = 592, *p* < 0.001, Fig 5A). Tuna collected in 2009–2014 had more prey in their stomachs on average (*n* = 34.4) than those collected in 2008 or 2015–2016 (*n* = 22.7 and 20.1, respectively, Games-Howell, *t* = 3.64, 7.76, *p* < 0.01). Individual stomachs contained a maximum of 161 *P*. *planipes* (265.9 g) in 2015 and 131 *E*. *mordax* (528.5 g) in 2016.

There were significant differences in the mean individual energetic values of prey among CART groups (Welch’s ANOVA on ranks, *F* = 9.59, *n* = 592, *p* < 0.001; Fig 5B). Individual prey had higher energetic values in 2015–2016 than in either 2008 or 2009–2014 (Games-Howell, *t* = 3.64, 3.84, *p* < 0.001). The total energetic value (kJ) of prey in a single stomach was also different among CART groups (Welch’s ANOVA on ranks, *F* = 6.19, *n* = 592, *p* = 0.003, Fig 5C). However only the energetic values of stomach contents in 2008 (median = 116.43 kJ) were significantly different from contents in 2009–2014 (median = 193.29 kJ, Games-Howell, *t* = 3.23, *p* = 0.005). There was no difference in the total energetic contents between stomachs collected in 2015–2016 (median = 124.24 kJ) and either of the other two CART groups (Games-Howell, *t* = 1.34, 2.29, *p* = 0.37, 0.06, Fig 5C). The total energetic values of stomach contents estimated with our methods were within the range of reported daily caloric intake of captive *T*. *orientalis* [45].

### Feeding behaviors

**Fig 6:**
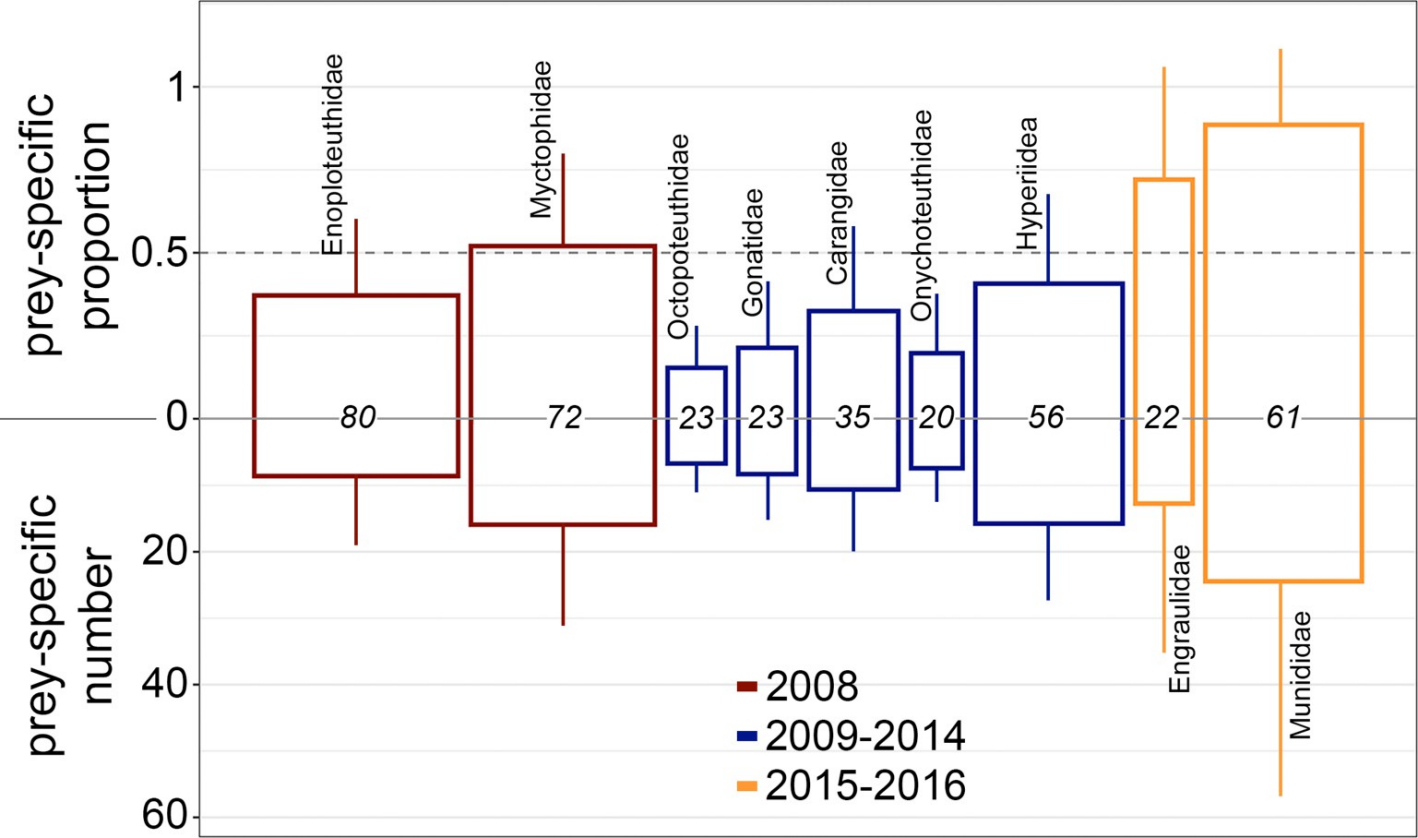
Feeding behaviors on prey found in at least 20% of *Thunnus orientalis* stomachs per CART group. For each prey, vertical polygon dimensions describe the mean prey-specific proportion (up from the x-axis) and mean prey-specific number (down from the x-axis) for which standard deviations are represented by whiskers. The frequency of occurrence of each prey per CART group corresponds to the width of the polygons and is given as text on the x-axis.

Using prey-specific proportion to estimate feeding behaviors of *T*. *orientalis*, we observed variability in the degree and subject of forage specialization among CART groups (Fig 6). In 2008, *T*. *orientalis* specialized on Myctophidae (prey-specific proportion > 0.5), with an average of 15.9 individuals per stomach (prey-specific number). *T*. *orientalis* sampled in 2009–2014 exhibited more generalist feeding (prey-specific proportion < 0.5) across a relatively diverse prey assemblage. In 2015–2016 *T*. *orientalis* specialized on *E*. *mordax* or *P*. *planipes*, though the smaller *P*. *planipes* were more frequently consumed and in higher numbers (mean prey-specific abundances of 12.7 and 24.5, respectively).

## Discussion

### Diet summary

*T*. *orientalis* forage on a broad diversity of fishes, squids, and crustaceans in the SCB, including high energy epipelagic prey such as anchovies and sardines, as well as large numbers of mesopelagic prey such as myctophids, hyperiid amphipods, and enoploteuthid squids. While our findings differ from the two previous studies of the *T*. *orientalis* diet in the SCB [10, 19], enoploteuthid squids are also important prey for smaller *T*. *orientalis* (< 30 cm FL) in the western Pacific Ocean [23]. Studies of similar sized bluefin from the Mediterranean (*T*. *thynnus*) and southwestern Pacific Ocean (*T*. *maccoyii*) reported similarly diverse diets, including mesopelagic species associated with deep-scattering layers [12, 35]. Prey items observed in this study were generally smaller than 10 cm, consistent with prey lengths of *T*. *maccoyii* in the southwestern Pacific [12] and other tuna species at similar sizes [46].

An increase in predator size often translates into foraging on larger items [47], but we did not observe variability in prey length with increasing *T*. *orientalis* mass (Table 2 and S3 Fig). Consumption of relatively small prey has been reported across a broad size range for bluefin and other tunas from multiple ocean basins [11, 12, 46]. Within the sampling periods defined in this study, *T*. *orientalis* ranging in mass from 2 to 103 kg (42–172 cm FL) foraged on similar sized prey (< 10 cm, < 10 g) at similar trophic levels (e.g. [48]). This is consistent with the findings of Madigan *et al*. (2017) who showed no trend in *δ*^*15*^*N* stable isotope values (a proxy for trophic position) in *T*. *orientalis* from ~70–200 cm FL in the CCS. Smaller, lower trophic level prey tend to have higher cumulative biomass [49] and may respond more quickly to environmental variability than higher trophic level prey. Targeting diverse, relatively small prey may increase the resilience of *T*. *orientalis* in a dynamic system, where forage composition and abundance can change drastically over relatively short timescales.

### Thunnus orientalis exhibits a flexible foraging ecology in the Southern California Bight

*T*. *orientalis* in this study were classified into three sampling periods based on prey taxonomic composition, with limited overlap in the dominant prey species. Additionally, prey consumed during these three sampling periods also had different size structures and behaviors. In 2008, *T*. *orientalis* fed mostly on medium sized (3–5 cm) myctophids and enoploteuthid squids, abundant constituents of the daytime deep scattering layer in the southern CCS (~300–500 m depth) [50–52] that migrate into near-surface waters at night [53, 54]. Archival tag data and diet analyses (where time of feeding was inferred from prey digestion state) suggest that bluefin tunas can feed during day and night [12, 35]. Although we do not know at what time of day the prey in this study were consumed, both day- and nighttime depth distributions of myctophids and enoploteuthid squids are within the diving range of *T*. *orientalis* (~ 0 to > 450 m) [5, 10].

In 2015–2016, *T*. *orientalis* fed heavily on *P*. *planipes* and *E*. *mordax* (3–8 cm), both of which can form dense schools in near-surface waters [55, 56]. These prey were often found in large numbers in stomachs containing little else (Fig 6), suggesting *T*. *orientalis* exploits such aggregations. Specialization on abundant, near-surface schooling prey most closely resembles the findings of Pinkas (1971, *E*. *mordax*) and Madigan et al. (2015, *S*. *sagax*). However, this strategy is not limited to epipelagic prey as *T*. *orientalis* also appeared to specialize on myctophids in 2008 when using a prey-specific proportion threshold of 0.5 (Fig 6, [10, 44]).

Prey consumed in 2009–2014 were the smallest (1–5 cm), most taxonomically diverse, and were consumed in the highest numbers. It is unlikely the increased diversity was simply due to a longer sampling period as each individual stomach had relatively high diversity (Fig 3A and S3 Table). The most abundant forage species also had diverse depth habitats, including the epipelagic Pacific jack mackerel, (*Trachurus symnmetricus)* [57], and the more broadly mesopelagic gonatid squids (Gonatidae) [53] and hyperiid amphipods (Hyperiidea) [58]. In contrast to the periods of prey specialization observed in this and previous studies, *T*. *orientalis* could be described as a generalist predator during 2009–2014, feeding throughout a broader swath of the water column in the SCB. This generalist foraging has also been observed in *T*. *thynnus* and *T*. *maccoyii* [12, 35]. Young et al (1997) found that *T*. *maccoyii* in nearshore and offshore habitats fed on diverse, but distinct diets that were correlated to differences in prey availability between habitats. While we were unable to directly compare diets to estimates of forage availability, each sampling period was characterized by prey with different depth habitats and schooling behaviors. Thus, it is likely that *T*. *orientalis* employs different feeding behaviors to opportunistically exploit these ecologically distinct forage.

### Diet variability coincides with responses of forage to climatic variability in the CCS

Although we were not able to quantify the effects of environmental conditions on diet variability in our dataset, temporal variability in *T*. *orientalis* foraging ecology coincides with major oceanographic and biological variability in the SCB. During the late 2007 to early 2009 La Niña event, the SCB was characterized by relatively cool sea surface temperatures, accompanied by low to neutral anomalies of phytoplankton and zooplankton biomass [59]. At the same time, relatively high abundances of larval myctophids were also observed [17], suggesting a large spawning biomass that could have been exploited by *T*. *orientalis*. The same surveys also noted low abundances of larval anchovy and sardine in 2008. ENSO neutral conditions prevailed from 2011–2014 following brief El Niño and La Niña events in 2009–2010 and 2010–2011, respectively. Consistently positive chlorophyll-*a* anomalies and variable water column temperatures during this period supported an order of magnitude variability in zooplankton biomass [60] and diverse larval fish assemblages, from which anchovy and sardine were notably absent [61]. Increased sea surface temperatures and stratification in the SCB during a 2014–2015 marine heat wave and an El Niño event in 2015–2016 were associated with relatively high abundances of anchovy [61]. During these warming events, the diets of multiple predators became dominated by anchovy (e.g. California sea lions (*Zalophus californianus*), rhinoceros auklet (*Cerorhinca monocerata*), [16]) and pelagic red crabs (yellowtail jack (*Seriola dorsalis*, *pers*. *communication and observation*), reflecting concerted food web changes across the CCS.

While these shifts were apparent in the diets of bluefin, little is known about the relationship between *T*. *orientalis* diet and metrics of forage availability in the CCS, the most extensive of which are derived from trawl surveys (e.g. CalCOFI and NOAA’s Coastal Pelagic Survey [16, 17]). Direct comparisons of predator diets to these trawl surveys are an important step in determining how well trawl estimates of forage availability reflect forage use by predators. For managed forage species (e.g. anchovy, sardine, rockfishes (*Sebastes spp*.)), predator diet monitoring could refine estimates of natural mortality under different environmental conditions. Ultimately, it will be important to continue diet analyses across seasons and climatic regimes (e.g. El Niño, La Niña) to more directly link predator diets to forage availability and oceanography variability.

### Feeding on diverse forage provides similar meal energetic value

Among sampling periods, there was limited variability in estimated meal energetic value (kJ per stomach, Fig 5C) despite differences in prey composition, sizes, and numbers (Figs 2, 4 and 5A). There was no difference in the total energetic value or number of prey per stomach when *T*. *orientalis* was specializing on myctophids in 2008 or *P*. *planipes* and *E*. *mordax* in 2015–2016 (Fig 6). However, the mean prey-specific number of *E*. *mordax* (12.7) was lower than Myctophidae (15.9), and the standard deviations of prey-specific numbers were higher in 2015–2016 than 2008. Myctophid aggregations have lower average densities and are larger on average (0.2–200 individuals m^-3^, 100s of m diameter by 10s of m thick [62–64]) than schools of *E*. *mordax* (0.5–500 individuals m^-3^, ~10s of m diameter x 20 m thick) [56, 65]. Myctophids may represent more consistent patches of prey across larger areas than *E*. *mordax* or *P*. *planipes*, which could explain their higher frequency of occurrence and lower variance in prey specific number (Fig 6) [56, 66, 67]. Stomachs collected in 2009–2014 contained prey with relatively low individual energetic values but had similar total energetic values to stomachs collected in 2015–2016 due to the high numbers of prey (Fig 6). Daily caloric intake is determined by the identity and number of prey consumed, but net energetic gains are tied to energy expenditure during search and capture [68].

*T*. *orientalis* exhibits diverse diving behaviors across seasons and habitats that have been tied to foraging [6, 7] and likely require different amounts of energy. Foraging near the surface on relatively high-density prey may have lower energetic costs than foraging throughout the water column on more diffusely distributed prey [69]. Additionally, the metabolic rate of juvenile *T*. *orientalis* may be elevated while foraging in relatively cool water at depth, outside of their metabolic thermal minimum zone of 15- 20°C [70]. Thus, prey consumed in 2009–2014 may have been more costly to capture than red crabs and anchovies in 2015–2016 and resulted in lower net energetic gains or growth rates. Madigan *et al*. [71] reported reduced growth rates in *T*. *orientalis* that fed higher proportions of midwater prey, although the influence of tuna size on this observation remains unclear. Future diet and archival tagging studies that can more directly link feeding and expenditures to predator condition or growth rate will elucidate the net value of foraging on these distinct prey groups.

### Implications for our understanding of bluefin tuna ecology and fisheries

**Fig 7:**
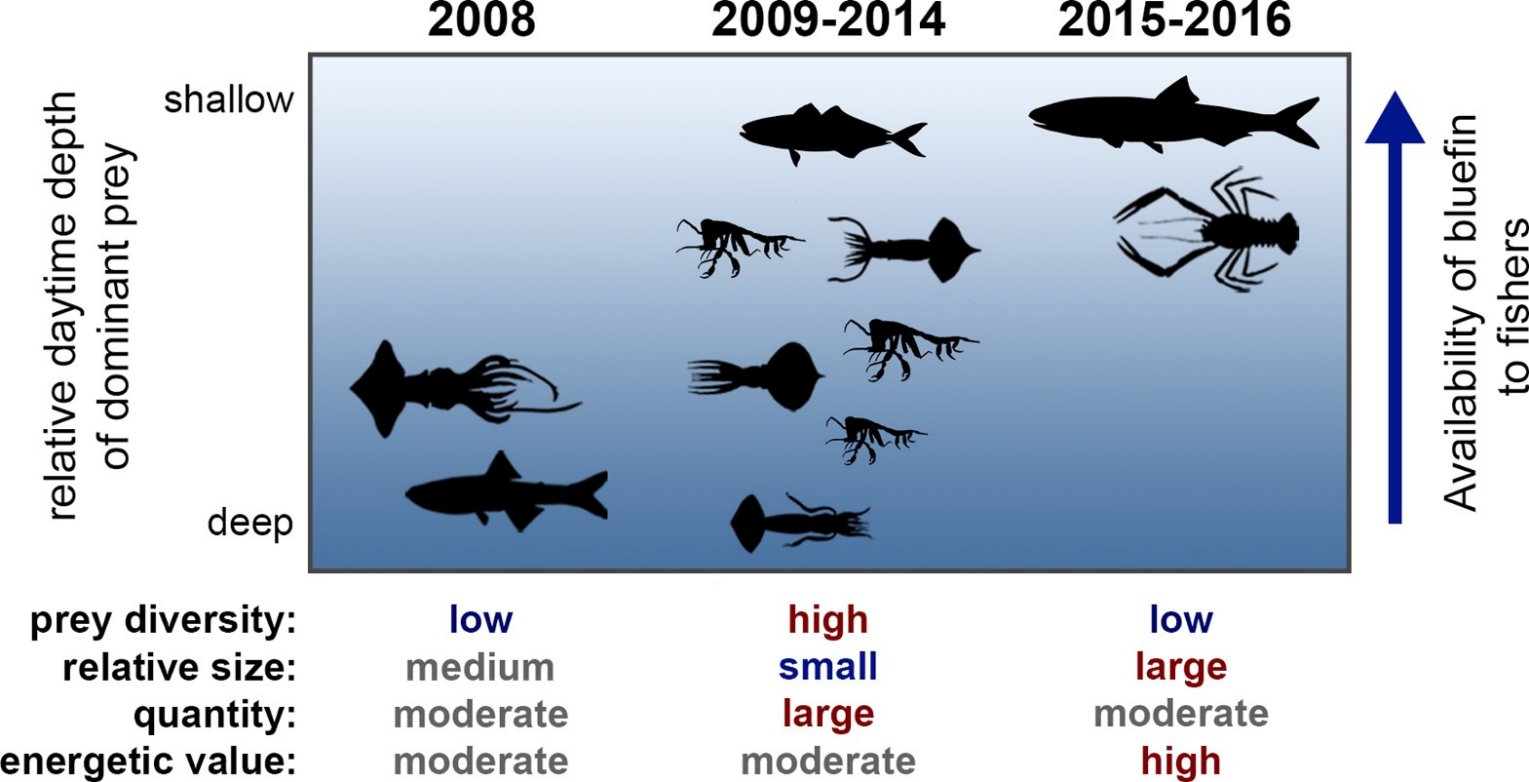
Relationship between stomach contents of *Thunnus orientalis* and its availability to fishers in the SCB. Silhouettes describe the identity and relative daytime depth of dominant prey families. The relative diversity, size, quantity, and energetic value of prey are also given for each sampling period.

Globally, bluefin tunas are often associated with energetically dense, schooling forage [11, 12, 19]. The distribution of forage fishes impacts the migration and density of tuna schools, and the availability of high energy forage is positively correlated with tuna condition and landings [6, 8, 13, 72]. Although it is not clear how prey switching affected its horizontal distribution or abundance in the SCB, daytime depth distributions of *T*. *orientalis* significantly impact its availability to recreational fishers, which largely operate during daytime. When considering the daytime depths of dominant prey across the sampling periods defined in this study, the daytime depth of *T*. *orientalis* likely shoaled throughout our study period (Fig 7). Recreational fishers had a relatively difficult time hooking *T*. *orientalis* in 2008–2014, when fish were only found at depths from 50 to 150 meters, compared to 2015–2016 when fish were consistently observed in surface waters jumping and boiling on bait balls (*pers*. *communication and observation)*. Understanding the foraging ecology of *T*. *orientalis* can help to explain differential effort required to capture tunas.

## Conclusions

We present the most extensive time series of *T*. *orientalis* feeding ecology to date and describe a more flexible diet than previously observed in the CCS. *T*. *orientalis* is an opportunistic forager that exhibits periods of specialization and more generalist feeding, demonstrating the importance of multi-year diet studies to capture the dynamics of foraging ecology. This flexible feeding ecology has likely contributed to the persistence of *T*. *orientalis* in the CCS in the face of significant ecological variability over the past two decades. Continued diet monitoring that explicitly links diet to oceanographic conditions will improve our understanding of the drivers of variability in *T*. *orientalis* foraging ecology and may increase our capacity to predict changes in its distribution and availability to fishers.

## Supporting information

S1 Fig Pearson’s correlation coefficients among covariates considered in the CART analysis. (TIF)

S2 Fig Partial effects plots of the generalized additive model describing the effects of (a) estimated *Thunnus orientalis* mass and (b) sampling year on total prey mass per stomach. Full model results are given in Table 2. Each panel shows the relationship between a covariate and the contribution of the smoother for that covariate to the model’s fitted values (“s(x)”). Grey shading indicates 95% confidence intervals about the estimate for each covariate, and rug plots indicate covariate observations.
(TIF)

S3 Fig Partial effects plots of the generalized additive model describing the effects of (a) estimated *Thunnus orientalis* mass, (b) sampling year, and (c) prey group on prey length. Full model results are given in Table 2. Panels (a) and (b) show the relationship between a covariate and the contribution of the smoother for that covariate to the model’s fitted values (“s(x)”). Grey shading indicates 95% confidence intervals about the estimate for each covariate, and rug plots indicate covariate observations. In panel (c), the effect of prey group on prey length (“f(x)”) is given in reference to the mean length of cephalopods and error bars indicate 95% confidence intervals about each group mean.
(TIF)

S1 Table Summary of samples per year indicating the number of *Thunnus orientalis* stomachs and minimum number of sampling events per year. (DOCX)

S2 Table Regressions used to obtain prey lengths, masses, and energetic values from prey measurements. The equation and definition of all variables are given for each regression. The references (“ref”) are also provided.
(DOCX)

S3 TableMean proportional abundance ($\bar{p}$) and percent frequency of occurrence (%FO) of prey groups included in the CART analysis for each terminal node (“CART group”).“Other” groups include prey identified at least to family that represented less than 1% ($\bar{p}$) across the dataset (indicated by asterisks in Table 1).(DOCX)

S4 Table Sample coverage and diversity estimates for *Thunnus orientalis* stomachs in each CART group. *N* = the total number of stomachs per year group and *t* = the sample size at 99% sample coverage. Diversity estimates using the Shannon Index are given at 99% sample coverage (*qD* ± 95% confidence interval).
(DOCX)

S1 Data (XLSX)
