# Supplementary figures and images for: Pacific bluefin tuna, Thunnus orientalis, exhibits a flexible feeding ecology in the Southern California Bight
Source: PLoS One. 2022 Aug 25;17(8):e0272048. doi: 10.1371/journal.pone.0272048 (PMC9409590; doi:10.1371/journal.pone.0272048)

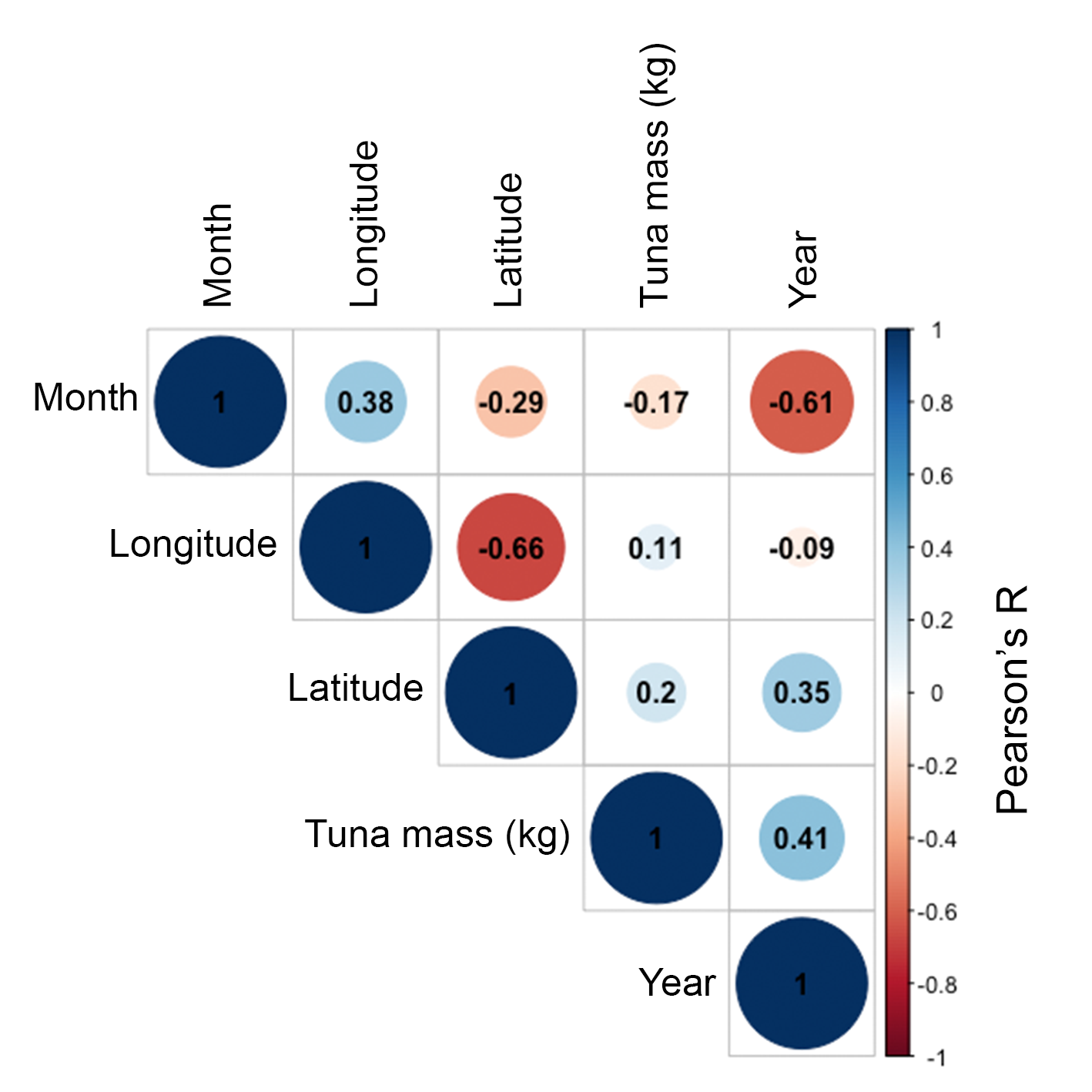

Supplement: S1 Fig — (TIF) [file pone.0272048.s001.tif]

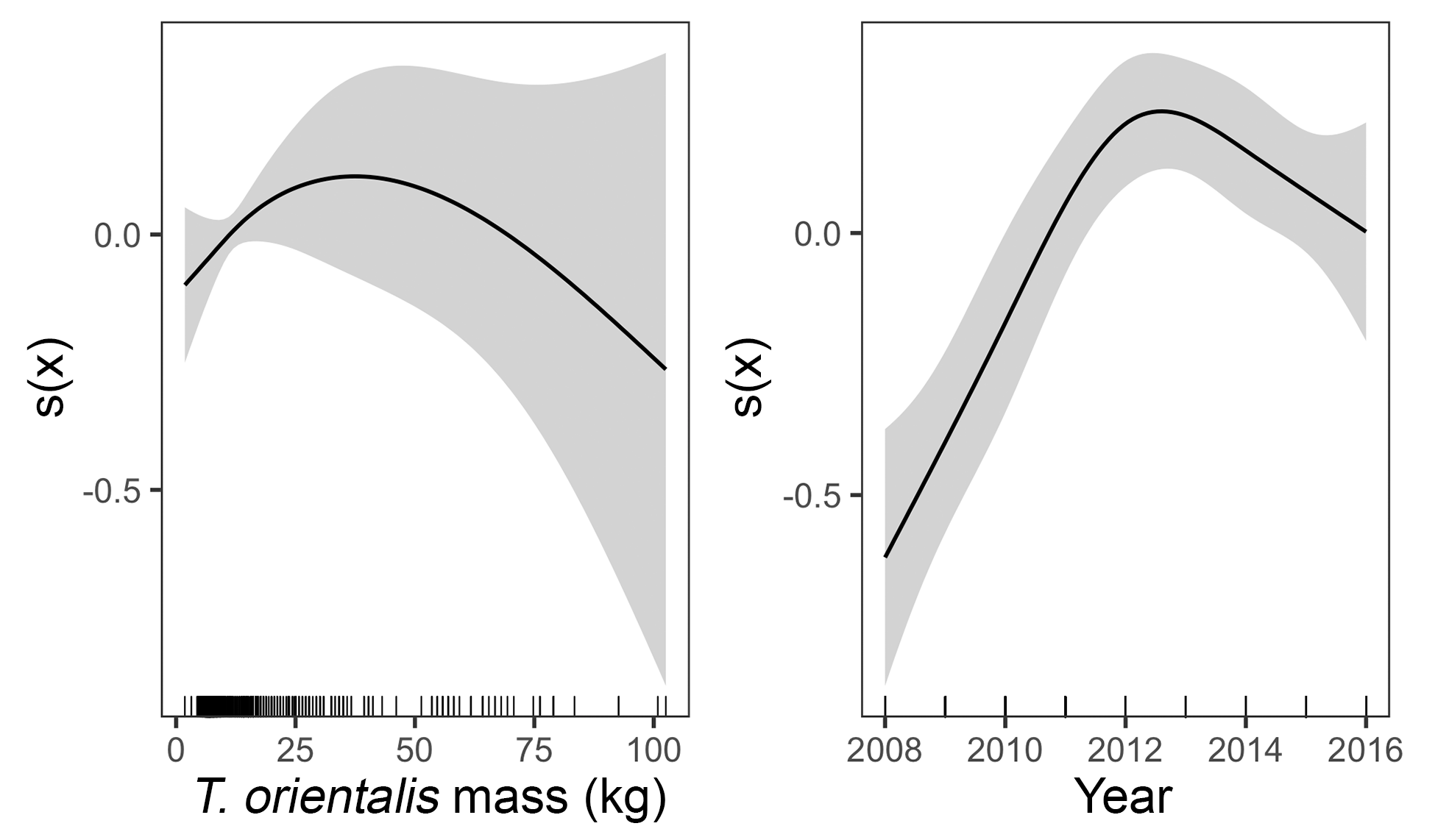

Supplement: S2 Fig — Partial effects plots of the generalized additive model describing the effects of (a) estimated Thunnus orientalis mass and (b) sampling year on total prey mass per stomach. Full model results are given in Table 2. Each panel shows the relationship between a covariate and the contribution of the smoother for that covariate to the model’s fitted values (“s(x)”). Grey shading indicates 95% confidence intervals about the estimate for each covariate, and rug plots indicate covariate observations. (TIF) [file pone.0272048.s002.tif]

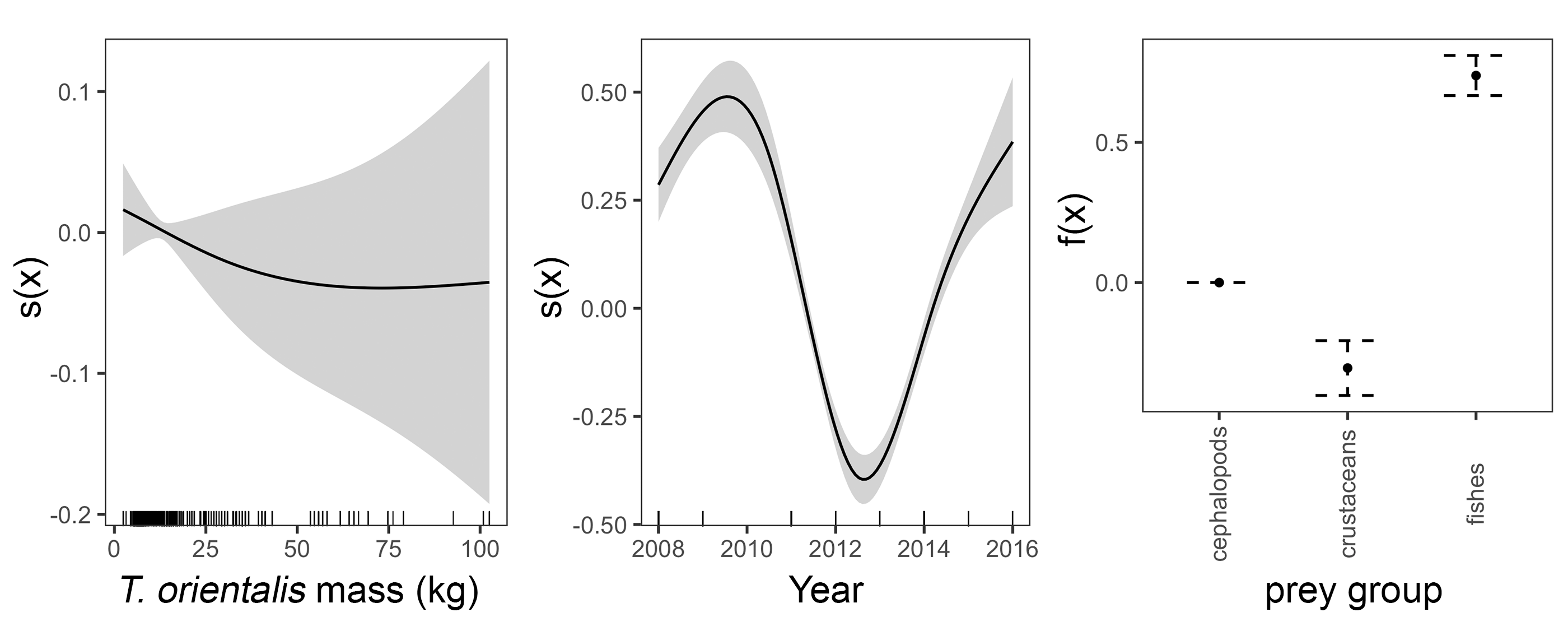

Supplement: S3 Fig — Partial effects plots of the generalized additive model describing the effects of (a) estimated Thunnus orientalis mass, (b) sampling year, and (c) prey group on prey length. Full model results are given in Table 2. Panels (a) and (b) show the relationship between a covariate and the contribution of the smoother for that covariate to the model’s fitted values (“s(x)”). Grey shading indicates 95% confidence intervals about the estimate for each covariate, and rug plots indicate covariate observations. In panel (c), the effect of prey group on prey length (“f(x)”) is given in reference to the mean length of cephalopods and error bars indicate 95% confidence intervals about each group mean. (TIF) [file pone.0272048.s003.tif]
