# Supplementary material for: Pacific bluefin tuna, Thunnus orientalis, exhibits a flexible feeding ecology in the Southern California Bight
Source: PLoS One. 2022 Aug 25;17(8):e0272048. doi: 10.1371/journal.pone.0272048 (PMC9409590; doi:10.1371/journal.pone.0272048)
Supplement: S1 Table — (DOCX) [file pone.0272048.s004.docx]

| **Year** | **Stomachs (with prey)** | **Min. number of sampling events/trips** |
| --- | --- | --- |
| 2008 | 88 | 13 |
| 2009 | 57 | 3 |
| 2010 | 45 | 3 |
| 2011 | 85 | 20 |
| 2012 | 93 | 19 |
| 2013 | 80 | 12 |
| 2014 | 79 | 11 |
| 2015 | 123 | 30 |
| 2016 | 71 | 27 |
