# Supplementary material for: Pacific bluefin tuna, Thunnus orientalis, exhibits a flexible feeding ecology in the Southern California Bight
Source: PLoS One. 2022 Aug 25;17(8):e0272048. doi: 10.1371/journal.pone.0272048 (PMC9409590; doi:10.1371/journal.pone.0272048)
Supplement: S2 Table — The equation and definition of all variables are given for each regression. The references (“ref”) are also provided. (DOCX) [file pone.0272048.s005.docx]

|  |  | **Length (mm)** | | | **Weight (g)** | | | | **Energy density (kJ/g)** | |
| --- | --- | --- | --- | --- | --- | --- | --- | --- | --- | --- |
|  |  | equation | *a* | ref | equation | *a* | *b* | ref | *a* | ref |
| **Family** | **Prey ID** | y = a*x |  |  | y = a*x^b |  |  |  |  |  |
|  |  |  |  |  |  |  |  |  |  |  |
| ***Fishes*** |  |  |  |  |  |  |  |  |  |  |
| Argentinidae | *Argentina sialis* | x = VCL (cm)  y = SL (cm) | 1.1 | Glaser 2010 | x = TL (cm)  y = BW (g) | 0.003 | 3.306 | Fishbase Argentina sialis | 6.58* | see unidentified |
| Carangidae | *Trachurus symmetricus* | x = VCL (cm)  y = SL (cm) | 1.1 | Glaser 2010 | x = SL (cm)  y = BW (g) | 0.0635 | 2.556 | Harvey 2000 | 6.4 | Glaser 2010 |
| Centrolophidae | *Icichthys lockingtoni* | x = VCL (cm)  y = SL (cm) | 1.1 | Glaser 2010 | x = SL (cm)  y = BW (g) | 0.024725 | 3.025 | Family median, fishbase | 6.58* | see unidentified |
| Clupeidae | *Sardinops sagax* | x = VCL (cm)  y = SL (cm) | 1.3 | Glaser 2010 | x = SL (cm)  y = BW (g) | 0.00763 | 3.15 | fishbase | 7.3 | Glaser 2010 |
| Engraulidae | *Engraulis mordax* | x = VCL (cm)  y = SL (cm) | 1.1 | Glaser 2010 | x = SL (cm)  y = BW (g) | 0.0485 | 2.413 | Harvey 2000 | 6.36 | Sidwell 1981 |
| Exocoetidae | *Cheliopogon pinnatibarbatus* | x = VCL (cm)  y = SL (cm) | 1.1 | Glaser 2010 | x = SL (cm)  y = BW (g) | 0.01191375 | 3.01875 | see unidentified | 6.58* | see unidentified |
| Labridae | *Oxyjulis californica* | x = VCL (cm)  y = SL (cm) | 1.1 | Glaser 2010 | x = SL (cm)  y = BW (g) | 0.019555 | 3.0 | Family median, fishbase | 6.58* | see unidentified |
| Merlucuciidae | *Merluccius productus* | x = VCL (cm)  y = SL (cm) | 1.1 | Glaser 2010 | x = SL (cm)  y = BW (g) | 0.0081 | 2.966 | Harvey 2000 | 4.96 | Mean from Abitia-Cardenas et al 1997 and Sidwell 1981 |
| Microstomatidae | *Nansenia* sp. | x = VCL (cm)  y = SL (cm) | 1.1 | Glaser 2010 | x = SL (cm)  y = BW (g) | 0.01191375 | 3.01875 | see unidentified | 10.42 | Nansenia from Sinclair et al 2015 |
| Myctophidae | *Ceratoscopelus townsendi* | x = VCL (cm)  y = SL (cm) | 1.1 | Glaser 2010 | x = SL (cm)  y = BW (g) | 0.007155 | 3.11865 | Family median, fishbase | 3.56 | Childress et al 1980 *C. warmingii |
| Myctophidae | *Diaphus theta* | x = VCL (cm)  y = SL (cm) | 1.1 | Glaser 2010 | x = SL (cm)  y = BW (g) | 0.007155 | 3.11865 | Family median, fishbase | 10.33 | Mean from Sinclair et al 2015 and Childress and Nygaard 1973 |
| Myctophidae | *Nannobrachium ritteri* | x = VCL (cm)  y = SL (cm) | 1.1 | Glaser 2010 | x = SL (cm)  y = BW (g) | 0.007155 | 3.11865 | Family median, fishbase | 6.95 | *Lampanyctus ritteri from Childress and Nygaard 1973 |
| Myctophidae | *Protomyctophum crockeri* | x = VCL (cm)  y = SL (cm) | 1.1 | Glaser 2010 | x = SL (cm)  y = BW (g) | 0.007155 | 3.11865 | Family median, fishbase | 5.94 | Sinclair et al 2015 |
| Myctophidae | *Stenobrachius leucopsaurus* | x = VCL (cm)  y = SL (cm) | 1.1 | Glaser 2010 | x = SL (cm)  y = BW (g) | 0.00451 | 3.31 | Fishbase | 8.64 | Mean from Sinclair et al 2015 and Childress and Nygaard 1973 |
| Myctophidae | *Symbolophorus californiensis* | x = VCL (cm)  y = SL (cm) | 1.1 | Glaser 2010 | x = SL (cm)  y = BW (g) | 0.007155 | 3.11865 | Family median, fishbase | 4.26 | * similar water content to T. crenularis Childress and Nygaard 1973 |
| Myctophidae | *Tarletonbeania crenularis* | x = VCL (cm)  y = SL (cm) | 1.1 | Glaser 2010 | x = SL (cm)  y = BW (g) | 0.007155 | 3.11865 | Family median, fishbase | 4.26 | Childress and Nygaard 1973 |
| Myctophidae | *Triphoturus mexicanus* | x = VCL (cm)  y = SL (cm) | 1.1 | Glaser 2010 | x = SL (cm)  y = BW (g) | 0.00354 | 3.35 | Fishbase | 7.03 | Mean from Childress and Nygaard 1973 and Sidwell 1981 |
| Myctophidae | Myctophidae | x = VCL (cm)  y = SL (cm) | 1.1 | Glaser 2010 | x = SL (cm)  y = BW (g) | 0.007155 | 3.11865 | Family median, fishbase | 6.67 | Mean of all unique Myctophid values used here (n=7) |
| Ophidiidae | *Chilara taylori* | x = VCL (cm)  y = SL (cm) | 1.1 | Glaser 2010 | x = SL (cm)  y = BW (g) | 0.0004 | 3.761 | Harvey 2000 | 6.58* | see unidentified |
| Paralepididae | *Lestidiops ringens* | x = VCL (cm)  y = SL (cm) | 1.1 | Glaser 2010 | x = SL (cm)  y = BW (g) | 0.01191375 | 3.01875 | see unidentified | 6.58* | see unidentified |
| Paralepididae | *Magnisudis atlantica* | x = VCL (cm)  y = SL (cm) | 1.1 | Glaser 2010 | x = SL (cm)  y = BW (g) | 0.01191375 | 3.01875 | see unidentified | 6.58* | see unidentified |
| Pleuronectidae | *Pleuronichthys decurrens* | x = VCL (cm)  y = SL (cm) | 1.1 | Glaser 2010 | x = SL (cm)  y = BW (g) | 0.00112 | 3.25 | Family median, fishbase | 4.78 | Mean from Spitz et al 2010 and Sidwell 1981 |
| Pleuronectiformes | Pleuronectiformes | x = VCL (cm)  y = SL (cm) | 1.1 | Glaser 2010 | x = SL (cm)  y = BW (g) | 0.00112 | 3.25 | Family median, fishbase | 4.78 | Mean from Spitz et al 2010 and Sidwell 1981 |
| Sciaenidae | *Seriphus politus* | x = VCL (cm)  y = SL (cm) | 1.1 | Glaser 2010 | x = SL (cm)  y = BW (g) | 0.0207 | 2.98 | Family median, fishbase | 6.58* | see unidentified |
| Scomberesocidae | *Cololabis saira* | x = VCL (cm)  y = SL (cm) | 1.2 | Glaser 2010 | x = KL* (mm) **  y = BW (g) | 0.000001653 | 3.172 | Median from Hughes 1974 | 7.5 | Glaser 2010 |
| Scombridae | *Scomber japonicus* | x = VCL (cm)  y = SL (cm) | 1.2 | Glaser 2010 | x = SL (cm)  y = BW (g) | 0.10 | 3.10 | Furuichi et al, 2021 | 6.69 | Mean from Abitia-Cardenas et al 1997, Sidwell 1981, and Eder and Lewis 2005 |
| Scopelarchidae | *Rosenblattichthys volucris* | x = VCL (cm)  y = SL (cm) | 1.1 | Glaser 2010 | x = SL (cm)  y = BW (g) | 0.01191375 | 3.01875 | see unidentified | 6.58* | see unidentified |
| Sebastidae | *Sebastes* spp. | x = VCL (cm)  y = SL (cm) | 1.1 | Glaser 2010 | x = SL (cm)  y = BW (g) | 0.0287 | 2.985 | Family median, fishbase | 4.2 | Glaser 2010 |
| Syngnathidae | *Syngnathus californiensis* | x = VCL (cm)  y = SL (cm) | 1.1 | Glaser 2010 | x = SL (cm)  y = BW (g) | 0.01191375 | 3.01875 | see unidentified | 6.58* | see unidentified |
|  | Unidentified fishes | x = VCL (cm)  y = SL (cm) | 1.1 | Glaser 2010 | x = SL (cm)  y = BW (g) | 0.01191375 | 3.01875 | Median of all regressions used of form y = a*x^b | 6.58 | Mean of all unique energy density values referenced here (n=16) |

- * KL = knob length. Used SL as KL was not available for our specimens.
- Family median from fishbase
  - Subset for family of interest and regressions built using Standard Lengths (SL) for which values of *a* and *b* are present.
- Median of all regressions used of form y = a*x^b
  - Using all regressions (except *Cololabis* from Hughes because KL in mm), but only one for each family so as not to skew median calculation towards fish identified with relatively high taxonomic resolution (only used single entry of *a* and *b* for Myctophidae and Plueronecti(dae/formes))

**Table S2 continued:** For prey for which regressions could not be found, the identity of an appropriate analog that was used is indicated with an asterisk.

|  |  | **Length (mm)** | | | | **Weight (g)** | | | | **Energy density (kJ)** | | |
| --- | --- | --- | --- | --- | --- | --- | --- | --- | --- | --- | --- | --- |
|  |  | type | *a* | *b* | ref | type | *a* | *b* | ref | | *a* | ref |
| **Family** | **Prey ID** |  |  |  |  |  |  |  |  | |  |  |
|  |  |  |  |  |  |  |  |  |  | |  |  |
| ***Cephalopods*** | |  |  |  |  |  |  |  |  | |  |  |
| Amphitretidae | *Japetella heathi* | x = BW(g)  y=ML (mm)  y = (x/a)^b | 9.97 E^-05^ | 0.3355705 | Schwarz et al 2020 | UHL (mm) to BW(g)  y = (x/a)^b | 22.08 | 0.7042254 | Schwarz et al 2020 | | 0.32 | Chen et al In Prep |
| Argonautidae | *Argonauta* sp.  **Ocythoe tuberculata* | UHL(mm) to ML(mm)  y = a*x+b | 4.47 | 0.83 | Lu and Ickeringill 2002 | UHL (mm) to BW (g)  y = x^a/(e^b) | 2.67 | 2.14 | Lu and Ickeringill 2002 | | 3.8 | *Octopus sp. from  Meynier et al 2008 |
| Enoplotethidae | *Abraliopsis affinis* | LRL (mm*) to ML (mm)  y = a*x+b | 19.28 | 9.8 | Wolff 1984 | LRL (cm) to BW (g)  y = x^a*e^b | 2.1 | 5.5 | Wolff 1984 | | 4.02 | Chen et al In Prep |
|  | *Abraliopsis felis* | LRL (mm*) to ML (mm)  y = a*x+b | 40.55 | -2.66 | Wolff 1984 | LRL (cm) to BW (g)  y = x^a*e^b | 2.49 | 6.58 | Wolff 1984 | | 4.02 | Chen et al In Prep |
| Gonatidae | *Gonatopsis* sp.  **Gonatopsis borealis* | LRL (mm) to ML (mm)  y = a*x+b | 38.14 | 2.11 | Sinclair et al 2015 | ML (mm) to BW (g)  Y = ax^b | 7.142 E^-05^ | 2.872 | Sinclair et al 2015 | | 4.20 | Mean from *Gonatopsis sp.*  Perez 1994  and Sinclair et al 2015 |
|  | *Gonatus* sp.  **Gonatus* *middendorfi* | LRL (mm) to ML (mm)  y = a*x+b | 47.51 | 1.72 | Sinclair et al 2015 | ML (mm) to BW (g)  Y = ax^b | 1.39 E^-04^ | 2.552 | Sinclair et al 2015 | | 5.95 | Mean from *Gonatus sp.*  Lawson et al 1998  and Sinclair et al 2015 |
|  | *Gonatus* sp.  onyx | LRL (mm*) to ML (mm)  y= a*x+b | 19.02 | 12.82 | Wolff 1984 | LRL (cm) to BW (g)  y = x^a*e^b | 2.13 | 4.99 | Wolff 1984 | | 5.95 | Mean from *Gonatus sp.*  Lawson et al 1998  and Sinclair et al 2015 |
| Histioteuthidae | *Histioteuthis heteropsis* | LRL (mm*) to ML (mm)  y = a*x+b | 20.57 | 2.04 | Wolff 1984 | LRL (cm) to BW (g)  y = x^a*e^b | 2.64 | 7.43 | Wolff 1984 | | 2.65 | Clarke et al 1985 |
| Loliginidae | *Doryteuthis opalescens* | LRL (mm) to ML (mm)  y = a*x+b | 60.78 | 32.4 | Wolff 1984* was loligo | LRL (cm) to BW (g)  y = x^a*e^b | 1.4 | 6.0 | Wolff 1984* was loligo | | 3.26 | *Loligo vulgaris  Sidwell 1981 |
| Octopodidae | *Octopus bimaculatus*  **Octopus kaurna* | UHL (mm) to ML (mm)  y = a*x+b | 18.54 | 0.72 | Lu and Ickeringill 2002 | UHL (mm) to BW (g)  y = x^a*e^b | 2.77 | 1.14 | Lu and Ickeringill 2002 | | 3.8 | *Octopus sp. from  Meynier et al 2008 |
|  | *Octopus rubescens*  ** Octopus kaurna* | UHL (mm) to ML (mm)  y = a*x+b | 18.54 | 0.72 | Lu and Ickeringill 2002 | UHL (mm) to BW (g)  y = x^a*e^b | 2.77 | 1.14 | Lu and Ickeringill 2002 | | 3.8 | *Octopus sp. from  Meynier et al 2008 |
| Octopoteuthidae | *Octopoteuthis* sp. | LRL (mm) to ML (mm)  y = a*x+b | 18.55 | -1.51 | Lu and Ickeringill 2002 | LRL (cm) to BW (g)  y = x^a*e^b | 2.54 | 0.23 | Lu and Ickeringill 2002 | | 3.03 | Clarke et al 1985 |
| Ommastrephidae | *Dosidicus gigas* | LRL (mm) to ML (mm)  y = a*x+b | 35.79 | 44.2 | Wolff 1984 | LRL (cm) to BW (g)  y = x^a*e^b | 2.48 | 7.4 | Wolff 1984 | | 6.57 | Abitia-Cardenas et al 1997 |
| Onychoteuthidae | *Onychoteuthis borealijaponica* | LRL (mm) to ML (mm)  y = a*x+b | 50.99 | -19.893 | Lowry et al 2020 | LRL (mm) to BW (g)  y = ax^b | 1.11255 E ^-04^ | 2.7555 | Median of male and female from Bigelow 1994 | | 5.4 | Perez 1994 |
| Decapodiformes | Unidentified squids | LRL (mm) to ML (mm) | 36.965 | 2.075 | Median of all squid regressions used of form  y = a*x+b | LRL (cm) to BW (g) | Need to use median of values that come out of regressions because units of x and equations are variable | | | | 4.39 | Mean of unique regressions  For squid listed here |
|  |  |  |  |  |  |  |  |  |  | |  |  |
| ***Crustaceans*** | |  |  |  |  |  |  |  |  | |  |  |
| Euphausiidae | Euphausiidae | TL measured | - | - | constant | - | - | 0.08 | * median value for  Euphausiid of approximately 20mm TL  Kulka 1982 | | 3.11 | Davis et al 1998 |
| Hyperiidea | *Phronima* sp. | TL measured | - | - | constant | - | - | 0.1 | * median value for  Phronima from Bishop and Geiger 2006 | | 2.46 | Davis et al 1998 |
|  | Oxycephalidae |  |  |  |  |  |  |  |  | |  |  |
|  | Hyperiidea | TL measured | - | - | constant | - | - | 0.1 | * median value for  Phronima from Bishop and Geiger 2006 | | 2.46 | Davis et al 1998 |
| Munididae | *Pleuroncodes planipes* | y = TL (mm)  x = CL (mm)  y = x*a | 2.45 | - | Boyd 1962 | CL (mm) to BW(g)  Y = ax^b | 0.00095 | 2.755 | Median from Rodriguez-Jaramillo et al 2018 | | 3.47 | Mean of values supplied in  Abitia-Cardenas et al 1997 and  Sidwell 1981 |
| Malacostraca | Malacostraca | - | - | - | constant | - | - | 0.1 | - | | 3.2 | Glaser 2010 |

**REFERENCES**

Abitia-Cardenas LA, Galvan-Magana F, Rodriguez-Romero J (1997) Food habits and energy values of prey of striped marlin, Tetrapturus audax, off the coast of Mexico. Fish Bull 95:360–368

Bigelow KA. Age and growth of the oceanic squid Onychoteuthis borealijaponica in the North Pacific. Fishery Bulletin. 1994 Jan 1;92(1):13-25.

Bishop, R. E., & Geiger, S. P. (2006). Phronima Energetics: Is There a Bonus to the Barrel? *Crustaceana*, *79*(9), 1059–1070. <http://www.jstor.org/stable/20107736>

Boettiger C, Temple Lang D, Wainwright P (2012). “rfishbase: exploring, manipulating and visualizing FishBase data from R.” Journal of Fish Biology. <https://doi.org/10.1111/j.1095-8649.2012.03464.x>.

Boyd, C. M. (1962). The Biology of a Marine Decapod Crustacean, Pleuroncodes planipes Stimpson, 1860. UC San Diego: Scripps Institution of Oceanography. Retrieved from https://escholarship.org/uc/item/80n8w92r

Childress JJ, Nygaard MH (1973) The chemical composition of midwater fishes as a function of depth of occurrence off Southern California. Deep-Sea Res 20:1093–1109

Childress JJ, Taylor SM, Cailliet GM, Price MH (1980) Pat- terns of growth, energy utilization and reproduction in some meso- and bathypelagic fishes off Southern California. Mar Biol 61:27–40

Clarke, A., Clarke, M., Holmes, L., & Waters, T. (1985). Calorific Values and Elemental Analysis of Eleven Species of Oceanic Squids (Mollusca:Cephalopoda). *Journal of the Marine Biological Association of the United Kingdom,* *65*(4), 983-986. doi:10.1017/S0025315400019457

Davis ND, Myers KW, Ishida Y (1998) Caloric value of high- seas salmon prey organisms and simulated salmon ocean growth and prey consumption. Annu Rep North Pac Anadromous Fish Comm 1:146–162

E. B. Eder & M. N. Lewis. (2005). Proximate composition and energetic value of demersal and pelagic prey species from the SW Atlantic Ocean. *Marine Ecology Progress Series*, *291*, 43–52.

Furuichi, S., Kamimura, Y. & Yukami, R. Length–length and Length–weight Relationships for Four Dominant Small Pelagic Fishes in the Kuroshio–Oyashio Current System. *Thalassas* **37,**651–657 (2021). https://doi.org/10.1007/s41208-021-00300-9

Glaser SM (2010) Interdecadal variability in predator–prey interactions of juvenile North Pacific albacore in the California Current System. Mar Ecol Prog Ser 414:209-221. <https://doi.org/10.3354/meps08723>

Harvey, J.T., Loughlin, T.R., Perez, M.A., and Oxman, D.S., Relationship between fish size and otolith length for 63 species of fishes from the Eastern North Pacific Ocean, in NOAA Technical Report NMFS No. 150, Seattle, WA, NOAA/Natl. Mar. Fish. Service, 2000, pp. 1–38.

Hughes, S.E. 1974. Stock composition, growth, mortality, and availability of Pacific saury, *Cololabis saira*, of the northeastern Pacific Ocean. Fishery Bulletin **22**(1): 121-131.

D. W. Kulka, S. Corey, Length and Weight Relationships of Euphausiids and Caloric Values of Meganyctiphanes Norvegica (M. Sars) in the Bay of Fundy, Journal of Crustacean Biology, Volume 2, Issue 2, 1 April 1982, Pages 239–247, <https://doi.org/10.2307/1548004>

Lawson JW, Magalhaes AM, Miller EH (1998) Important prey species of marine vertebrate predators in the northwest Atlantic: proximate composition and energy density. Mar Ecol Prog Ser 164:13–20

Lu, C. C., and Ickeringill, R. 2002. Cephalopod beak identification and biomass estimation techniques: tools for dietary studies of southern Australian finfishes. Museum Victoria Science Reports 6: 1–65. DOI: https://doi.org/10.24199/j.mvsr.2002.06

Mark S. Lowry, K. Alexandra Curtis, Christiana M. Boerger. 2020. Measurements and regressions of otoliths, cephalopod beaks, and other prey hard parts used to reconstruct California Current predator diet composition, U.S. Department of Commerce, NOAA Technical Memorandum NMFS-SWFSC-637.

L. Meynier , P. C. H. Morel , D. D. S. Mackenzie , A. Macgibbon , B. L. Chilvers & P. J. Duignan (2008) Proximate composition, energy content, and fatty acid composition of marine species from Campbell Plateau, New Zealand, New Zealand Journal of Marine and Freshwater Research, 42:4, 425-437, DOI: 10.1080/00288330809509971

Perez MA (1994) Calorimetry measurements of energy value of some Alaskan fishes and squids. Report No. NTIS No. PB94-152907

Rodríguez-Jaramillo, C., Cordoba-Matson, M. V., Zenteno-Savin, T., Balart, E. F., Méndez-Rodríguez, L. C., & De-Anda-Montañez, J. A. (2018). Reproductive Biology of the Red Crab Pleuroncodes planipes (Anomuran, Galatheid) on the West Coast of the Baja California Peninsula, Mexico. *Journal of Shellfish Research*, *37*(5), 1093-1102. https://doi.org/10.2983/035.037.0521

Richard Schwarz, Uwe Piatkowski, Bruce H. Robison, Vladimir V. Laptikhovsky, Henk-Jan Hoving. Life history traits of the deep-sea pelagic cephalopods Japetella diaphana and Vampyroteuthis infernalis. Deep Sea Research Part I: Oceanographic Research Papers, Volume 164, 2020, 103365,https://doi.org/10.1016/j.dsr.2020.103365.

Sidwell VD (1981) Chemical and nutritional composition of finfishes, whales, crustaceans, mollusks, and their prod- ucts. U.S. Dept. of Commerce, National Oceanic and Atmospheric Administration, National Marine Fisheries Service, Seattle, Washington

Sinclair EH, Walker WA, Thomason JR (2015) Body Size Regression Formulae, Proximate Composition and Energy Density of Eastern Bering Sea Mesopelagic Fish and Squid. PLoS ONE 10(8): e0132289. <https://doi.org/10.1371/journal.pone.0132289>

Jérôme Spitz, Emeline Mourocq, Valérie Schoen, Vincent Ridoux, Proximate composition and energy content of forage species from the Bay of Biscay: high- or low-quality food?, ICES Journal of Marine Science, Volume 67, Issue 5, July 2010, Pages 909–915, <https://doi.org/10.1093/icesjms/fsq008>

Wolff GA. Identification and estimation of size from the beaks of 18 species of cephalopods from the Pacific Ocean [Internet]. NOAA/National Marine Fisheries Service; 1984. Available from: http://hdl.handle.net/1834/20593
