# Supplementary material for: Pacific bluefin tuna, Thunnus orientalis, exhibits a flexible feeding ecology in the Southern California Bight
Source: PLoS One. 2022 Aug 25;17(8):e0272048. doi: 10.1371/journal.pone.0272048 (PMC9409590; doi:10.1371/journal.pone.0272048)
Supplement: S3 Table — “Other” groups include prey identified at least to family that represented less than 1% (p¯) across the dataset (indicated by asterisks in Table 1). (DOCX) [file pone.0272048.s006.docx]

|  | $\bar{p}$ | | | ***%FO*** | | |
| --- | --- | --- | --- | --- | --- | --- |
| ***CART group*** | **2008** | **2009-14** | **2015-16** | **2008** | **2009-14** | **2015-16** |
| **Cephalopods** |  |  |  |  |  |  |
| *Argonautidae* | *0.00* | *0.00* | *0.02* | *1.1* | *14.6* | *4.5* |
| *Enoplotethidae* | *0.27* | *0.00* | *0.00* | *77.3* | *0.2* | *2.2* |
| *Gonatidae* | *0.02* | *0.05* | *0.02* | *10.2* | *22.1* | *8.2* |
| *Loliginidae* | *0.01* | *0.02* | *0.04* | *5.7* | *14.6* | *9.0* |
| *Octopodidae* | *-* | *0.04* | *0.00* | *-* | *16.2* | *3.0* |
| *Octopoteuthidae* | *0.00* | *0.04* | *-* | *2.3* | *21.6* | *-* |
| *Onychoteuthidae* | *0.01* | *0.03* | *0.02* | *18.2* | *18.5* | *9.0* |
| *Other Cephalopods* | *0.02* | *0.00* | *0.00* | *13.6* | *5.5* | *0.7* |
| *Unididentified Squids* | *0.03* | *0.15* | *0.03* | *4.5* | *42.8* | *13.4* |
| **Fishes** |  |  |  |  |  |  |
| *Carangidae* | *0.01* | *0.11* | *0.02* | *1.1* | *33.0* | *8.2* |
| *Clupeidae* | *0.00* | *0.03* | *0.03* | *3.4* | *16.2* | *13.4* |
| *Engraulidae* | *0.01* | *0.02* | *0.14* | *3.4* | *5.5* | *30.6* |
| *Myctophidae* | *0.36* | *0.02* | *0.01* | *69.3* | *9.1* | *2.2* |
| *Scomberesocidae* | *0.01* | *0.03* | *0.00* | *3.4* | *13.0* | *3.7* |
| *Scombridae* | *-* | *0.04* | *0.00* | *-* | *18.0* | *1.5* |
| *Sebastidae* | *0.06* | *0.05* | *0.04* | *13.6* | *19.6* | *15.7* |
| *Other Fishes* | *0.00* | *0.03* | *0.02* | *3.4* | *10.0* | *13.4* |
| *Unidentified Fishes* | *0.04* | *0.03* | *0.06* | *26.1* | *17.5* | *17.9* |
| **Crustaceans** |  |  |  |  |  |  |
| *Hyperiidea* | *-* | *0.19* | *0.01* | *-* | *52.6* | *7.5* |
| *Munididae* | - | - | *0.52* | - | - | *84.3* |
| *Unidentified Malacostraca* | *0.15* | *0.06* | *0.04* | *59.1* | *20.3* | *12.7* |
