# Supplementary material for: Pacific bluefin tuna, Thunnus orientalis, exhibits a flexible feeding ecology in the Southern California Bight
Source: PLoS One. 2022 Aug 25;17(8):e0272048. doi: 10.1371/journal.pone.0272048 (PMC9409590; doi:10.1371/journal.pone.0272048)
Supplement: S4 Table — N = the total number of stomachs per year group and t = the sample size at 99% sample coverage. Diversity estimates using the Shannon Index are given at 99% sample coverage (qD ± 95% confidence interval). (DOCX) [file pone.0272048.s007.docx]

| **Group** | ***N*** | ***t*** | ***qD (± 95% CI)*** |
| --- | --- | --- | --- |
| 2008 | 88 | 88 | 8.57 (0.89) |
| 2009-2014 | 439 | 35 | 16.33 (0.37) |
| 2015-2016 | 194 | 133 | 11.45 (0.90) |
